# *Drosophila* Lipophorin Receptors Recruit the Lipoprotein LTP to the Plasma Membrane to Mediate Lipid Uptake

**DOI:** 10.1371/journal.pgen.1005356

**Published:** 2015-06-29

**Authors:** Míriam Rodríguez-Vázquez, David Vaquero, Esmeralda Parra-Peralbo, John E. Mejía-Morales, Joaquim Culi

**Affiliations:** Centro Andaluz de Biología del Desarrollo (CSIC-UPO-JA), Universidad Pablo de Olavide, Sevilla, Spain; University of Utah, UNITED STATES

## Abstract

Lipophorin, the main *Drosophila* lipoprotein, circulates in the hemolymph transporting lipids between organs following routes that must adapt to changing physiological requirements. Lipophorin receptors expressed in developmentally dynamic patterns in tissues such as imaginal discs, oenocytes and ovaries control the timing and tissular distribution of lipid uptake. Using an affinity purification strategy, we identified a novel ligand for the lipophorin receptors, the circulating lipoprotein Lipid Transfer Particle (LTP). We show that specific isoforms of the lipophorin receptors mediate the extracellular accumulation of LTP in imaginal discs and ovaries. The interaction requires the LA-1 module in the lipophorin receptors and is strengthened by a contiguous region of 16 conserved amino acids. Lipophorin receptor variants that do not interact with LTP cannot mediate lipid uptake, revealing an essential role of LTP in the process. In addition, we show that lipophorin associates with the lipophorin receptors and with the extracellular matrix through weak interactions. However, during lipophorin receptor-mediated lipid uptake, LTP is required for a transient stabilization of lipophorin in the basolateral plasma membrane of imaginal disc cells. Together, our data suggests a molecular mechanism by which the lipophorin receptors tether LTP to the plasma membrane in lipid acceptor tissues. LTP would interact with lipophorin particles adsorbed to the extracellular matrix and with the plasma membrane, catalyzing the exchange of lipids between them.

## Author Summary

In multicellular animals, nutrients and metabolites required for cell growth are distributed throughout the body by the blood circulation or in insects, by hemolymph. The uptake of these molecules by cells is tightly controlled to ensure the necessary coordination between cellular requirements and organismal homeostasis. Here we examine the mechanisms that mediate the cellular uptake of lipids in *Drosophila melanogaster*, a model organisms increasingly used in studies of metabolic homeostasis and its intersection with growth, aging and disease. In *Drosophila*, the majority of hemolymph lipids are carried in a lipoprotein particle named lipophorin. Lipid uptake in organs such as the ovaries or the imaginal discs is initiated by the expression of receptors of the LDLR family in the cell membrane. We show that these receptors bind with high affinity to a circulating lipoprotein named LTP, recruiting it to the cell surface. Surprisingly, LTP is not a major lipid carrier but instead catalyzes the transfer of lipids from lipophorin to cells. Our results improve our understanding of a central aspect of lipid metabolism in *Drosophila* and illustrate that although homologous proteins of the LDLR family play central roles in lipid uptake across phyla, the specific molecular mechanisms involved are diverse.

## Introduction

Lipids are continuously trafficked between tissues, from sites of absorption and synthesis to the organs that will utilize them. These transport routes must adapt to the changing metabolic status and developmental stage of the animal. Thus, during the feeding period of *Drosophila* larvae, a main pathway of lipid transport originates at the gut and delivers lipids to the fat body for storage. Imaginal discs also accumulate considerable amounts of neutral lipids during this stage. In contrast, under starvation and during the non-feeding pupal stage, lipids are mobilized from the fat body to support organismal growth and metabolism. Other main transport routes carry neutral lipids derived from the fat body to the muscles during flight and, in adult females, large amounts of lipids are also transferred to vitellogenic oocytes as an essential energy reserve [1–4]. How these routes are regulated and how lipids are targeted to particular tissues at specific developmental times is not well understood.

In insects, lipids are transported in hemolymph as lipoprotein particles, the most abundant being lipophorin, which carries about 95% of all hemolymph lipids in *Drosophila* [1,4]. Each particle contains a single copy of Apolipophorin I and Apolipophorin II, derived from the cleavage of a common precursor with homology to mammalian ApoB [5] and multiple lipid species, predominantly diacylglycerol (DAG) and phospholipids. Circulating lipophorin comes in contact with all tissues and cells, allowing for the potential exchange of lipids. Unfortunately, the mechanisms that mediate and regulate this exchange are only partially understood. Classic experiments demonstrated that lipophorin operates by a shuttle mechanism. Apolipophorin has a long half-life, calculated to exceed one day in some species [6], and each particle participates in multiple cycles of lipid loading and unloading in tissues without apparent degradation of the Apolipophorin moiety [7]. Biochemical and kinetic studies indicated that the interaction of lipophorin with cells is mediated through specific receptors [8–11]. At the molecular level, the best characterized are the lipophorin receptors of the Low Density Lipoprotein Receptor (LDLR) family, which were initially identified by their capacity to induce lipophorin endocytosis when overexpressed in a cell culture system [12]. In *Drosophila*, *lipophorin receptor 1* and *2* (*lpr1* and *lpr2*) are required for the uptake of neutral lipids in imaginal discs, oocytes and oenocytes [13,14]. However, *lpr1*, *lpr2* double mutant animals are viable and do not display significant changes in total neutral lipid content, suggesting that the major routes for lipid transport are not grossly disrupted. *Drosophila* lipophorin receptors promote lipid uptake by an endocytosis independent mechanism still poorly characterized [13]. Interestingly, these genes generate multiple, functionally diverse isoforms. Those containing a specific LDLR class A (LA) domain mediate neutral lipids uptake whereas the involvement of the remaining isoforms in lipid metabolism is unclear [13].

In insects, the exchange of lipids between lipophorin and tissues was shown to be facilitated by a circulating, low abundance, high density lipoprotein named Lipid Transfer Particle (LTP). Early in vitro studies showed that LTP had a surprising catalytic activity. It promoted the exchange of lipids, mostly DAG, between lipophorin particles of different densities and even between human LDL and insect lipophorins [15]. Additional experiments demonstrated that LTP also promoted the transfer of lipids between explanted tissues and purified lipophorin in vitro. In particular, transfer of lipids from the midgut to lipophorin and from lipophorin to the fat body and to ovaries was shown to be blocked by an anti-LTP antibody and resumed by the addition of purified LTP [16–19]. More recently, the genes coding for *apoLTP* in *Drosophila* and in *Bombyx mori* were identified and novel mutations isolated [4,20]. One of the most prominent phenotypes of *apoLTP* loss of function in *Drosophila* is the accumulation of neutral lipids in the gut, a phenotype similar to *apolipophorin* silencing [21] that demonstrates the essential role of LTP for loading lipophorin with lipids in enterocytes [4].

Here, we examine the molecular mechanisms that mediate the transfer of neutral lipids from lipophorin to imaginal discs and to ovaries. We identified LTP as a novel lipophorin receptor ligand. Our results indicate that recruitment of LTP to cell membranes mediated by the lipophorin receptors is a key event that initiates the transfer of neutral lipids to cells.

## Results

### LTP is a ligand for a subset of lipophorin receptor isoforms

To improve our understanding of the molecular mechanisms involved in lipophorin receptor-mediated lipid uptake, we decided to search for lipophorin receptor interacting proteins that could potentially participate in the process. To this end, we used an affinity purification strategy. We selected isoforms Lpr2E and Lpr2F as baits. Lpr2E mediates lipid uptake in imaginal discs and ovaries whereas Lpr2F, despite being 95.5% identical to Lpr2E, is inactive in this regard and was used as a negative control [13]. Both isoforms were tagged with TAP at the C-termini to facilitate purification [22], overexpressed in ovaries, a tissue of high lipid uptake activity, and affinity purified from ovary extracts. Proteins that differentially co-purified with Lpr2E compared to the control Lpr2F were identified by mass spectrometry (S1 Fig). To increase the probability to find interactors, we performed a second affinity purification experiment using Lpr2E and Lpr2F extracellular domains as baits instead of full-length proteins. In this experiment, we directed expression of the secretable baits to the fat body and purified the proteins from total larval extracts. Notably, in both experiments we identified the circulating lipoprotein LTP as a main Lpr2E interactor. In contrast, LTP was not isolated when Lpr2F was used as bait, either in the full-length or in the secretable form (S1 Fig).

**Fig 1:**
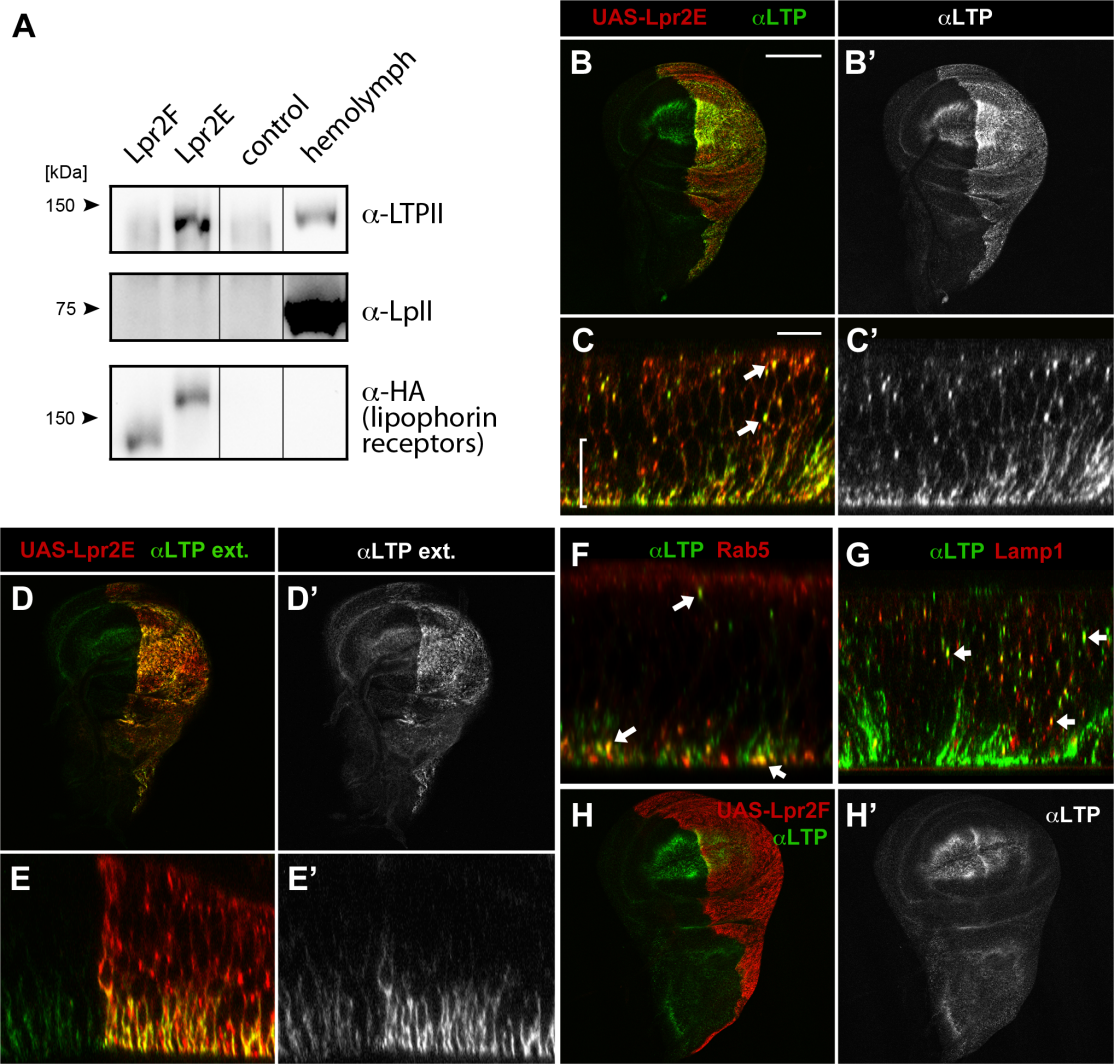
LTP is a ligand for a subset of lipophorin receptor isoforms. (A) Co-IP of LTP (top) and lipophorin (middle) with Lpr2F, Lpr2E or empty beads (control), analyzed by western blot. Lpr2E and Lpr2F, both containing an HA tag, were purified from transfected *Drosophila* S2 cells, incubated with diluted hemolymph and immunoprecipitated with anti-HA. Eluates were analyzed for the presence of lipophorin receptors, shown in the lower panel, of LTP, in the upper panel and of lipophorin, in the middle panel. Last lane contains 0.13 μl of diluted hemolymph. (B-E) Wing imaginal discs expressing *UAS-lpr2E* (red, detected with α-HA) in the posterior compartment driven by *hh-gal4*. Optical sections through the basal domain of imaginal discs (B and D) and cross-sections (C and E, apical domain at the top) are shown. LTP (green, also shown in a separate channel) accumulates at higher levels in the posterior compartment where Lpr2E is overexpressed (B and B'). In this region, LTP is detected at higher levels in basolateral membranes (bracket) as well as in vesicles (arrows), colocalizing with Lpr2E (C and C'). An immunostaining technique that solely detects extracellular proteins showed LTP (green, also in a separate channel) in basolateral membranes through the wing pouch area and at higher levels in the posterior compartment, where Lpr2E was overexpressed (D, D', E and E'). (F and G) Wing imaginal discs expressing *UAS-lpr2E* and *UAS-rab5-GFP* (F) or *UAS-lamp1-GFP* (G), shown in cross-section through the wing pouch area. LTP (green) is found in endocytic vesicles (arrows) partially colocalizing with Rab5 (red, F) and Lamp1 (red, G). (H) Wing imaginal disc expressing *UAS-lpr2F* (red) in the posterior compartment driven by *hh-gal4*. LTP distribution (green, also in a separate channel) imaged at a basal plane, is not modified by Lpr2F overexpression. B, D and H shown at the same magnification. Scale bar: 200 μm. C, E, F and G at the same magnification. Scale bar: 10 μm.

To validate the previous results, we examined the physical interaction between LTP and the lipophorin receptor isoforms by co-IP. HA tagged Lpr2E and Lpr2F isoforms were expressed and purified from *Drosophila* S2 cells and incubated in vitro with diluted hemolymph from wild type larvae. Hemolymph LTP strongly bound to Lpr2E, generating a robust signal, whereas it did not co-immunoprecipitate with Lpr2F (Fig 1A). Thus, these results confirm that LTP is a ligand of lipid uptake-promoting Lpr2E isoform but not of lipid uptake-inactive Lpr2F isoform and suggest that LTP might be a factor specifically involved in the transfer of lipids from lipophorin to tissues. Surprisingly, under the same conditions we were unable to detect an interaction between the lipophorin receptors and lipophorin (Fig 1A), even though lipophorin is more abundant than LTP in hemolymph. Thus, the affinity of lipophorin for the lipophorin receptors is weaker than that of LTP. Moreover, this result indicates that LTP binding to Lpr2E does not require lipophorin.

### Lipophorin receptors mediate LTP accumulation in ovaries and imaginal discs

Our in vitro data indicated Lpr2E binds with high affinity to LTP. To examine the functional relevance of this interaction, we first tested whether Lpr2E can promote LTP endocytosis or affect LTP distribution in vivo in imaginal discs, a tissue where lipophorin receptors activity was well characterized [13]. Interestingly, we found *UAS-lpr2E* overexpression induced a strong accumulation of LTP in basolateral cell membranes as well as formation of intracellular particles suggestive of LTP endocytosis (Fig 1B and 1C). These particles showed partial colocalization with the early endosome marker Rab5 and the lysosomal marker Lamp1, indicating that they represent different stages of LTP endocytosis (Fig 1F and 1G). The accumulation of LTP at the basolateral domain was mostly extracellular, since it could be detected with an immunostaining protocol performed without cell permeabilization (Fig 1D and 1E). Of notice, no such LTP accumulation or endocytosis was detected after Lpr2F overexpression (Fig 1H). Thus, isoform Lpr2E interacts in vivo with LTP and can promote LTP extracellular accumulation and endocytosis when overexpressed. The interaction is isoform-specific in vivo, as was already suggested by our in vitro data. In contrast, similar experiments indicated that overexpression of *UAS-lpr2E* or of *UAS-lpr2F*, both induced the endocytosis of lipophorin in imaginal discs, as shown by the formation of lipophorin intracellular vesicles that partially co-localized with endosome markers ([23] and S2 Fig). Thus, lipophorin receptor isoforms can induce lipophorin endocytosis irrespectively of their capacity to mediate lipid uptake.

**Fig 2:**
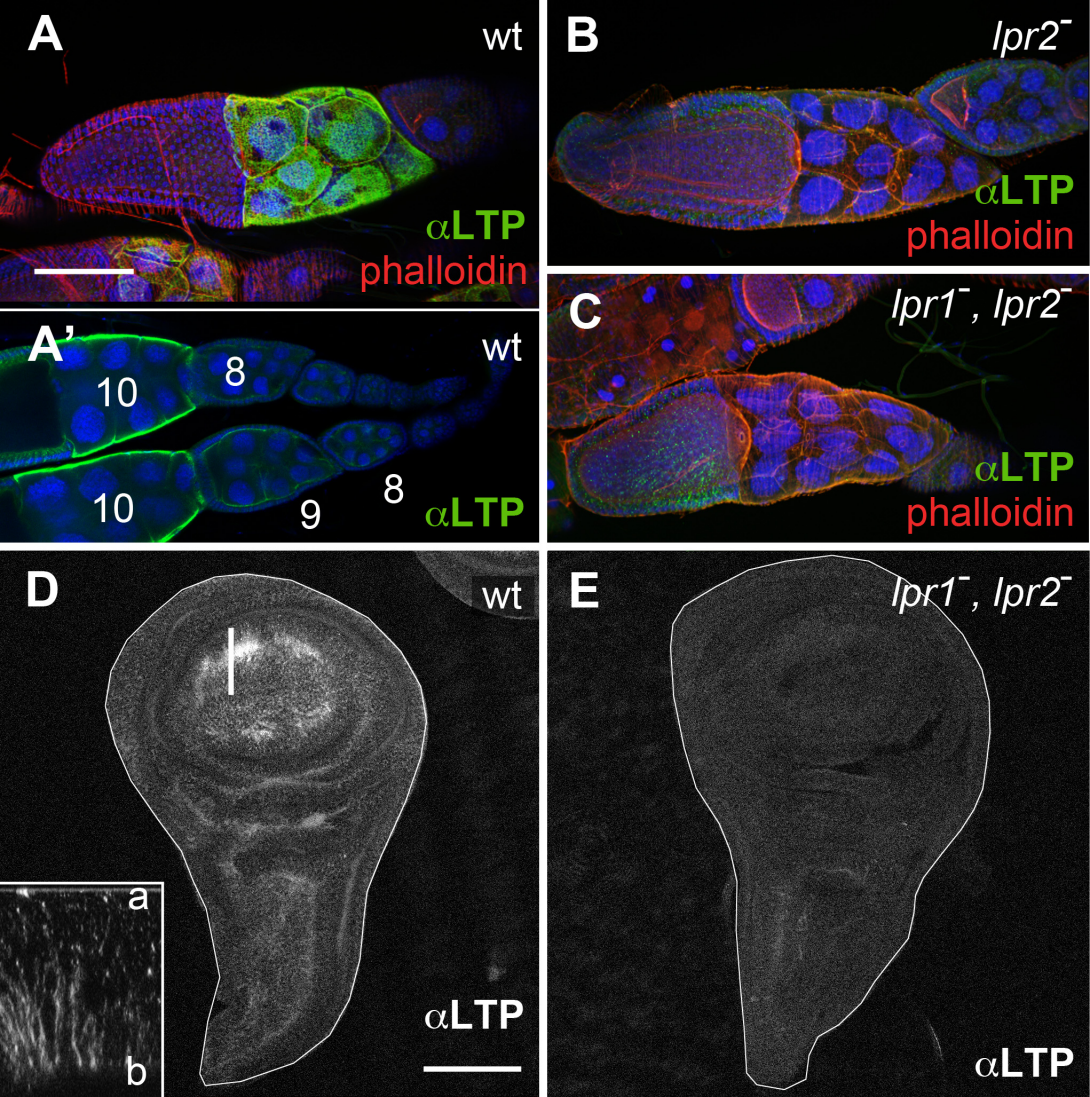
The lipophorin receptors are required for LTP accumulation in ovaries and imaginal discs. (A-C) Egg chambers of wild type (A), *Df(3R)lpr2* (B) and *Df(3R)lpr1/2* (C) genotypes, showing LTP distribution (green), F-actin (phalloidin, red) and nuclei (DAPI, blue). Images in A, B and C correspond to maximum intensity projections of approximately half egg chamber (50μm), whereas A' denotes a single optical plane to illustrate LTP accumulates at the surface of nurse cells. Stage 8, 9 and 10 egg chambers are labeled. LTP starts to significantly accumulate at stage 9 and peaks at stage 10. (D-E) Wing imaginal discs of wild type (D) and *Df(3R)lpr1/2* (E) genotypes showing LTP distribution. LTP is absent from the lipophorin receptor double mutant disc. Inset in D displays an optical cross-section (a: apical, b: basal) at higher magnification (4.5x) through the region of the disc marked with a bar. LTP mostly accumulates in basolateral membranes. Imaginal discs are outlined with a white line. A-C and D-E are shown at the same magnification. Scale bars: 100μm.

The lipophorin receptors are expressed in adult ovaries and in larval imaginal discs where they are required for neutral lipid uptake [13]. Thus, we examined whether LTP distribution in these tissues was altered in lipophorin receptor mutants. We found that in wild type ovaries, LTP accumulated in nurse cells plasma membranes starting at stage 9 follicles and strongly increasing at stage 10 (Fig 2A), essentially coinciding with *lpr2* expression pattern [13]. Interestingly, this accumulation mostly disappeared when *lpr2* was eliminated in *Df(3R)lpr2* or in *Df(3R)lpr1/2* females, which removes *lpr1* and *lpr2* genes (Fig 2B and 2C). In both mutants, occasional patches of LTP remained, mostly in crevices between nurse cells. Thus, *lpr2* is essential for LTP accumulation in nurse cells plasma membrane.

In wild type wing imaginal discs LTP is mostly found in the wing pouch region, accumulating in basolateral cell membranes as well as in a few apical vesicles (Fig 2D and inset). The wing pouch area expresses *lpr1* and *lpr2* and thus, these receptors could potentially mediate the observed LTP distribution. Accordingly, no LTP is detectable in imaginal discs from *lpr1*, *lpr2* double mutant larvae (Fig 2E). Taken together, our data demonstrates that both, in ovarian follicles and in imaginal discs cells, the lipophorin receptors are required for the accumulation of LTP at the cell surface. In addition, they mediate LTP endocytosis in imaginal discs.

We detected LTP in additional larval tissues. In particular, we saw a strong signal in gastric caeca and in discrete regions of the midgut (S3 Fig and [4]), the ring gland, the oenocytes, the salivary gland imaginal rings and a weaker staining in the fat body (S3 Fig). LTP distribution in these tissues did not change or slightly decreased in *Df(3R)lpr1/2* larvae (S3 Fig), suggesting the existence of other, still unidentified, LTP receptors.

### LTP is required for the accumulation neutral lipids in ovarian follicles and imaginal discs

**Fig 3:**
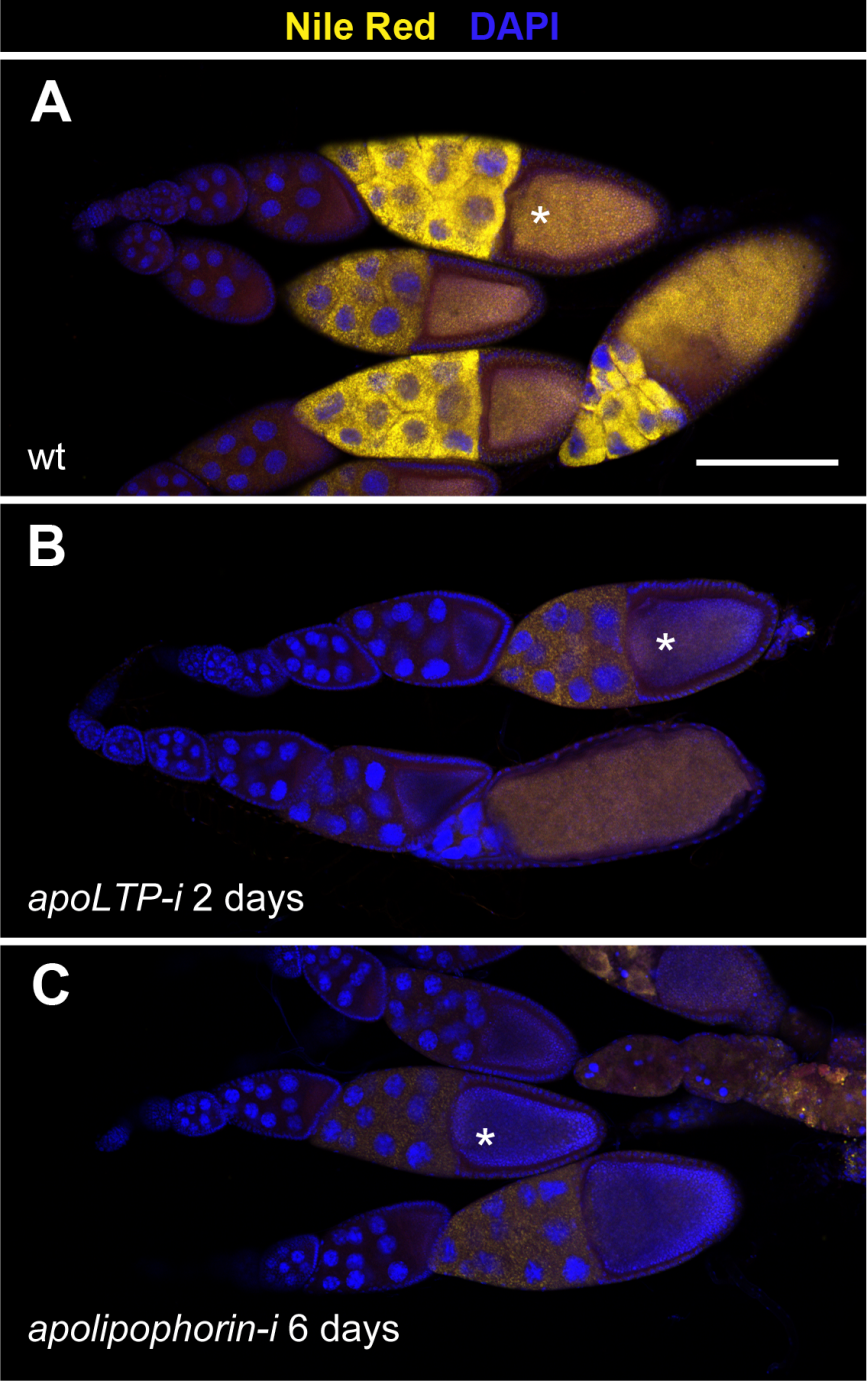
LTP is required for neutral lipid accumulation in ovaries. (A-C) Ovarioles containing egg chambers of progressive stages of development, with most mature egg chambers to the right. Neutral lipids are revealed by Nile red staining in yellow, nuclei (DAPI) in blue. Asterisks indicate vitellogenic egg chambers of an equivalent developmental stage (10b). (A) Wild type. (B) *apoLTP* was silenced in the fat body for two days prior dissection of the ovaries by the expression of *UAS-apoLTPi* driven by *Cg-gal4*. Temporal control was provided by a *tub-gal80* ^*ts*^ transgene. (C) *apolipophorin* was similarly silenced in the fat body for six days prior to dissection. Scale bar: 100μm.

The specific interaction between LTP and the lipophorin receptor isoforms that mediate lipid uptake suggests that LTP is involved in this process. To examine this possibility, we generated a novel mutation in *apoLTP* by the imprecise excision of the artificial transposon *P{wHy}DG06206*, inserted close to *apoLTP* promoter (S4 Fig). The excision removed a 4.8 Kb fragment which included the *apoLTP* promoter and the first non-coding exon, without affecting neighboring genes (S4 Fig). Accordingly, the novel mutation was named *apoLTP[excDG06206]*. In homozygous animals, embryogenesis was not affected. However, larvae remained small after hatching and eventually died after a prolonged first instar (S4 Fig, see also [4]). We observed a strong accumulation of neutral lipids in the gut of mutant larvae (S4 Fig), a phenotype also described by Palm et al. that reflects an essential role of LTP in the transfer of lipids from the gut to lipophorin [4]. The phenotype was caused by *apoLTP* loss of function since it could be completely rescued by a genomic BAC containing an *apoLTP* transgene. To examine *apoLTP* loss of function phenotype in adults, we silenced it in the fat body, the only tissue where the gene is expressed [4], by the temporally controlled expression of a *UAS-apoLTP-RNAi* transgene using the Gal80^ts^ technique [24] and the driver *Cg-gal4*, which is expressed in larval and adult fat body ([25] and S5 Fig). The most obvious phenotype was a pronounced reduction in female fertility two days after the activation of *UAS-apoLTP-RNAi*. In these animals, a fraction of the follicles degenerated. However, those that reached vitellogenic stages displayed strongly diminished levels of intracellular neutral lipids in nurse cells and oocytes (Fig 3A and 3B). Thus, LTP is required for the accumulation of neutral lipids by vitellogenic follicles. Silencing *apolipophorin* for six days using an equivalent approach resulted in a similar blockage of lipid uptake in ovarian follicles (Fig 3C). Thus, both lipophorin and LTP are required for the acquisition of neutral lipids during vitellogenesis.

To examine LTP requirement in imaginal discs, we silenced *apoLTP* in the fat body as before for four days. Since this treatment delays larval growth, we staged the animals by selecting white pupa, a developmental period that last for about one hour. *apoLTP* silenced pupa had imaginal discs of wild type size but notably reduced levels of neutral lipids compared to controls (S6 Fig and [4]), indicating that LTP is also required for lipid accumulation in imaginal discs.

Several hypotheses can account for the observed reduction in lipid droplets in ovaries and imaginal discs. LTP-lipopophorin receptor complexes could be required locally in these tissues for lipid uptake. Alternatively, the phenotypes could be indirectly caused by the blockage of lipophorin loading with lipids at the gut and the resulting decrease in lipid content of circulating lipophorin [4]. Finally, a combination of the two effects is also possible. However, the fact that LTP accumulates in nurse cells and imaginal disc cells plasma membranes and that only lipophorin receptor isoforms that mediate lipid uptake do induce this recruitment (Figs 1 and 2) support a direct involvement of LTP in the transfer of lipids from circulating lipophorin to nurse cells and imaginal disc cells.

### An extended LA-1 domain occurring in a subset of lipophorin receptor isoforms is essential for the interaction with LTP

**Fig 4:**
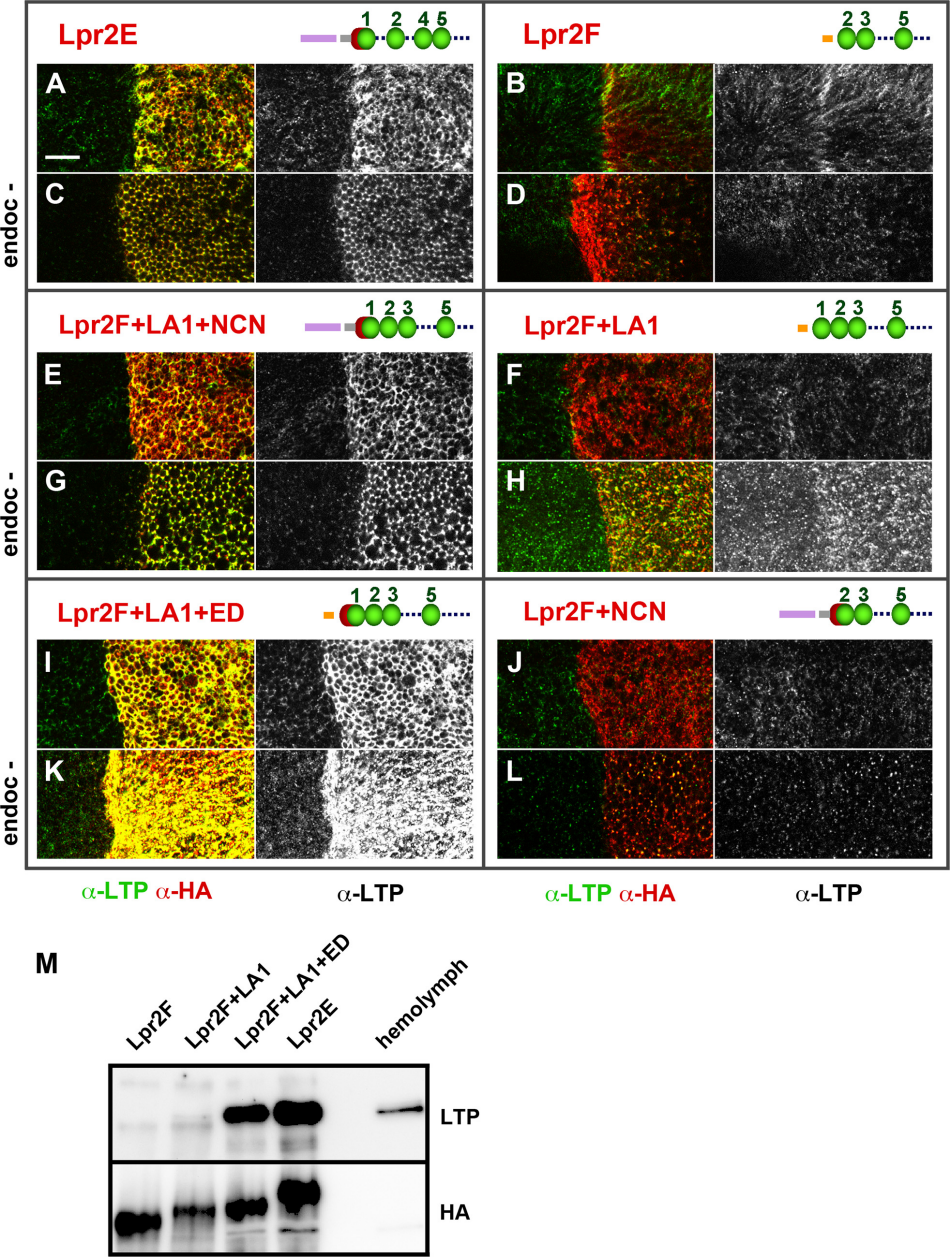
An extended LA-1 domain found in a subset of lipophorin receptor isoforms is required for robust LTP binding. (A-L) Wing imaginal discs shown at a basal plane, overexpressing in the posterior compartment the indicated lipophorin receptor isoforms and chimeras (α-HA, red). LTP distribution is shown in green and also in a separate channel. Panels marked as "endoc-" show imaginal discs in which endocytosis was blocked for three hours prior to dissection by inactivation of a temperature sensitive *shibire* allele (C, D, G, H, K and L). Drawings of the relevant N-terminal region of the receptors are included, the LA domains shown as green beads, the extension domain as a red half-sphere and Lpr2E and Lpr2F signal peptides as violet and orange bars respectively. The grey bar represents the non-conserved region of Lpr2E N-terminal domain. See (S7 Fig) for a more complete description of the chimeras used. All images are shown at the same magnification. Scale bar: 10μm. (M) Co-IP of LTP with several lipophorin receptor isoforms and chimeras, as indicated. Lpr2F, Lpr2F+A1, Lpr2F+A1+ED and Lpr2E were expressed and purified from *Drosophila* S2 cells and incubated with diluted wild type hemolymph. The lipophorin receptors and chimeras were immunoprecitated with α-HA and the eluates assayed by wester blot with α-LTP (upper panel) and α-HA (lower panel). Last lane contains hemolymph.

The observation that isoform Lpr2E physically interacted with LTP but isoform Lpr2F did not indicated that protein domains specifically present in Lpr2E were required for binding. To identify them, we assayed chimeric receptors generated by domain swapping between Lpr2E and Lpr2F (S7 Fig). It was previously shown that a 232 amino acids N-terminal region of Lpr2E was essential for neutral lipid uptake and for lipophorin extracellular stabilization (S7 Fig and [13]). Thus, we first tested whether the same N-terminal region played a role in LTP binding. To this end, we overexpressed the transgene *UAS-Lpr2F+LA1+NCN* coding for a chimera containing Lpr2E 232 amino acids N-terminal region fused to Lpr2F, in the posterior compartment of wing imaginal discs. This chimera induced LTP accumulation in basolateral plasma membranes as well as in intracellular vesicles, a phenotype identical to that of Lpr2E (compare Fig 4E and 4A). Thus, the 232 amino acids N-terminal domain of Lpr2E is required for the interaction with LTP. This region includes a specific LDLR A domain (LA-1) preceded by a stretch of 16 amino acids that is conserved between lipophorin receptors of several high dipteran species and that we call "extension domain" (ED), since it appears to extend the LA-1 domain (S7 Fig, underlined in red). In addition, Lpr2E and Lpr2F contain specific signal peptides. To examine which domains of the N-terminal region are involved in LTP binding, we tested additional chimeras in the same assay. Addition of LA-1 to Lpr2F (*UAS-lpr2F+LA1*) did not change the activity of the protein (Fig 4F, compare with Fig 4B). However, further addition of the 16 amino acids ED domain generating the chimera *UAS-lpr2F+LA1+ED*, provided a strong capacity to bind LTP (Fig 4I). In contrast, a similar chimera lacking LA-1 (*UAS-Lpr2F+NCN*) was unable to mediate LTP accumulation (Fig 4J). In conclusion, an LA-1 domain preceded by a stretch of 16 conserved amino acids (ED) is essential for a robust interaction between the lipophorin receptors and LTP.

A similar set of experiments was performed under conditions in which endocytosis was blocked for three hours before dissection using a temperature sensitive *shibire* (*shi*) allele, *Drosophila* Dynamin homolog. Under these conditions, receptors as well as their ligands accumulate at the cell surface improving their detection by immunostaining [26]. Consistent with our previous results, after blocking endocytosis we observed strong LTP extracellular accumulation in basolateral membranes for all isoforms and chimeras containing LA-1+ED domains (Fig 4C, 4G and 4K) and no effect on LTP distribution for Lpr2F or the chimera lacking LA-1 (Fig 4D and 4L). Interestingly, the chimera that contained LA-1 but not the ED (*UAS-lpr2F+LA1*) did promote a moderate LTP accumulation that was undetectable under normal conditions (Fig 4H). These results suggest that the LA-1 domain provides some capacity to bind LTP but the interaction is potentiated by the 16 conserved amino acids that precede it.

In vitro co-IP experiments examining the affinity between Lpr2F-Lpr2E chimeras and LTP gave results that were consistent with the previous in vivo data. We could not detect an interaction above background with Lpr2F (Fig 4M). Addition of the LA-1 module to Lpr2F conferred a very weak affinity. However, when the ED was also included, the interaction with LTP was robust and similar to that of Lpr2E (Fig 4M). Thus, ED synergizes with LA-1 to bind LTP.

In *Drosophila*, the extended LA-1 domain is present in five lipophorin receptor isoforms in addition to Lpr2E [13]. We tested two of them, Lpr1H and Lpr1J, for their capacity to interact with LTP and in both cases we saw LTP accumulation after overexpression in imaginal discs (S8 Fig). In contrast, no LTP stabilization was observed with isoform Lpr1M, which does not contain the extended LA-1 module (S8 Fig). Taken together, our results indicate that only Lpr1 and Lpr2 isoforms or chimeras containing the LA-1 module can bind LTP. Since the LA-1 module is also essential for the lipophorin receptors to mediate lipid uptake [13], our results strongly support a direct role of LTP-lipophorin receptor complexes in the cellular acquisition of lipids.

### LTP promotes a transient stabilization of lipophorin in the plasma membrane

**Fig 5:**
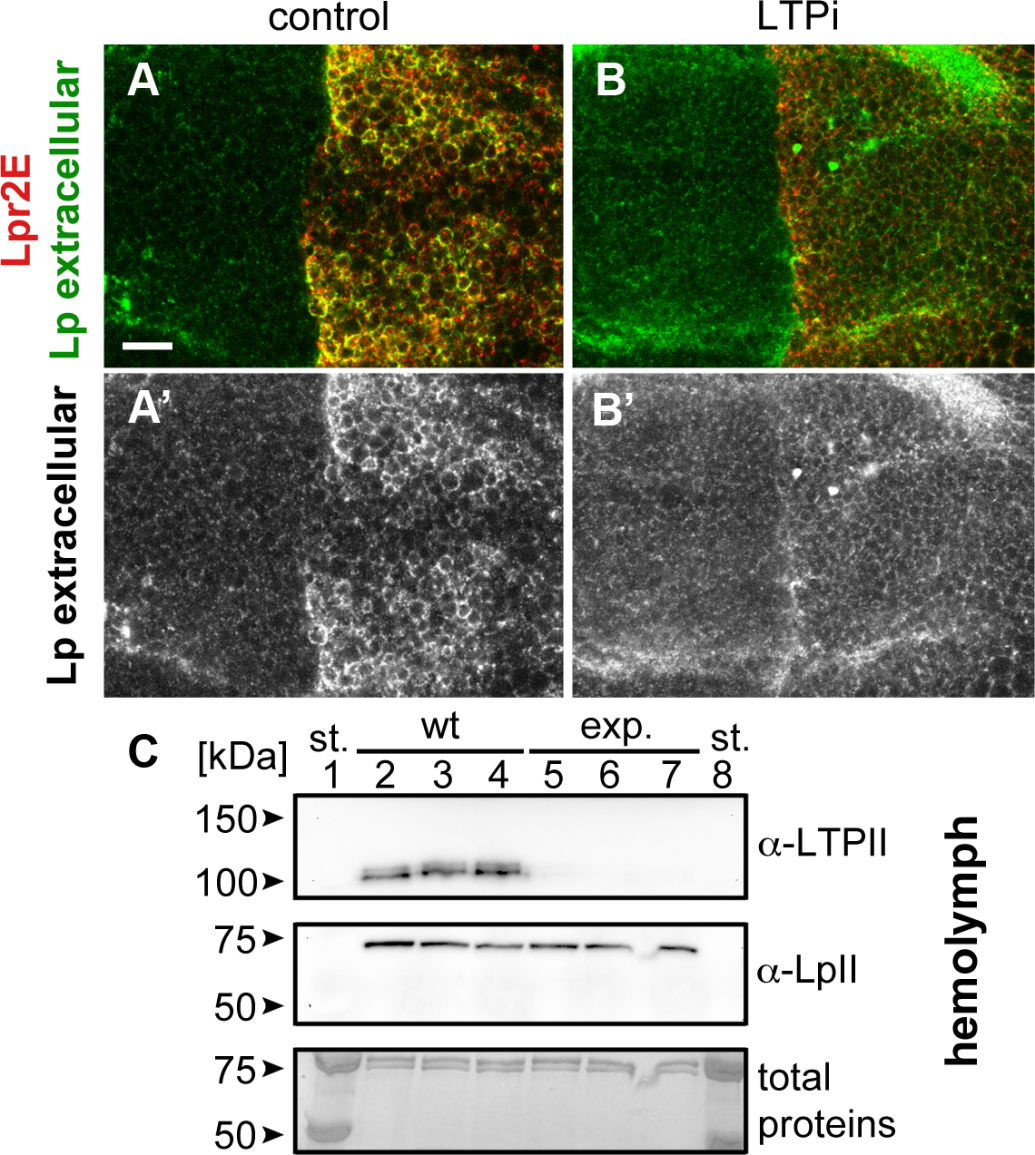
Lpr2E-mediated lipophorin extracellular stabilization requires LTP. (A and B) Wing imaginal discs expressing *UAS-lpr2E* in the posterior compartment, driven by *hh-gal4*. Lpr2E-HA is shown in red and extracellular lipophorin in green and also in a separate channel (A' and B'). (A) Control disc. (B) *apoLTP* was silenced by the expression of *UAS-apoLTPi* in the fat body driven by *Cg-gal4* for 2 days prior dissection. Temporal control was provided by a *tub-gal80* ^*ts*^ transgene. A strong reduction in lipophorin accumulation was observed. Note that for technical reasons, the complete genotype of *apoLTPi* animals shown in (B) was *Cg-gal4*,*tub-gal80* ^*ts*^/*UAS-Lpr2E*;*hh-gal4*/*UAS-apoLTPi*. Thus, *UAS-lpr2E* and *UAS-apoLTPi* were co-expressed both in the fat body and in imaginal discs. However, this does not have unintended effects, as shown in the next panel and in (S9 Fig). Scale bar: 10μm. (C) Western blot of hemolymph samples from wild type (wt, lanes 2–4) and the experimental animals (exp., lanes 5–7) described in (B). LTP and lipophorin were detected using the indicated antibodies. Three biological replicates were analyzed for each genotype. Total proteins stained with colloidal coomassie are shown as loading control at the bottom. Molecular weight markers were loaded in lanes 1 and 8. Circulating LTP levels are undetectable in the experimental animals whereas lipophorin levels are equivalent to the control.

**Fig 6:**
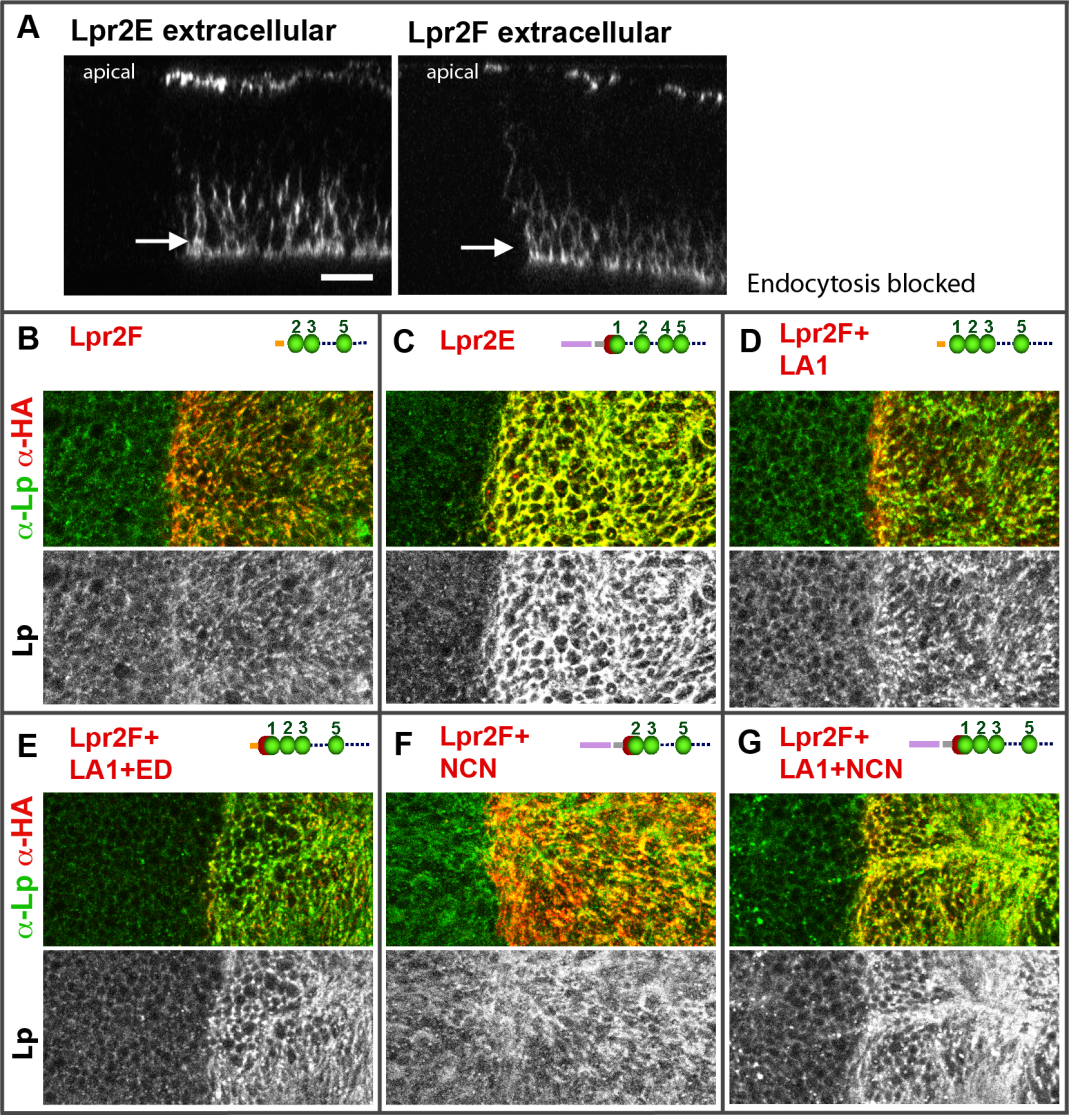
The LA-1 and ED domains of the lipophorin receptors are required for robust lipophorin stabilization in imaginal discs. (A) Optical cross-sections of wing imaginal discs expressing *UAS-lpr2E-myc* and *UAS-lpr2F-myc* in the posterior compartment, as indicated. Extracellular proteins were detected by an immunohistochemical protocol performed without cell permeabilization. Lpr2E and Lpr2F accumulate at similar levels in two domains, apical cell membranes and a basal region. (B-G) Wing imaginal discs expressing different lipophorin receptor isoforms and chimeras in the posterior compartment, as indicated. All images were taken at a basal plane of the imaginal discs. Its approximate location is indicated by arrows in A. Lipophorin (green and also in a separate channel) and the overexpressed lipophorin receptors and chimeras (red, detected with α-HA) are shown. All immunostainings were performed after blocking endocytosis for 2.5 hours. (A-G) Shown at the same magnification. Scale bar: 10μm.

**Fig 7:**
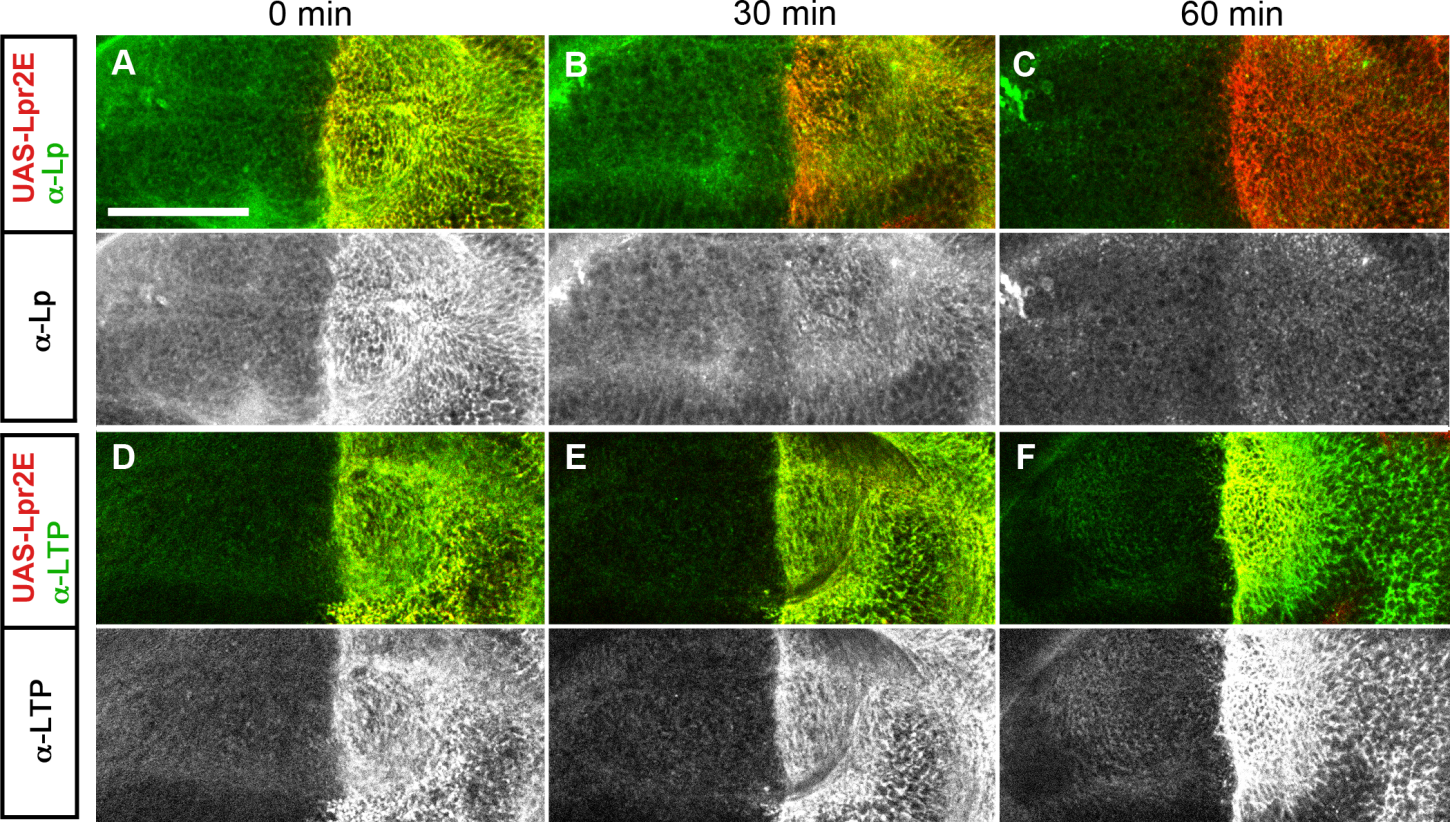
Lpr2E-mediated lipophorin association with cells is transient. (A-F) Basal optical sections of imaginal discs expressing *UAS-lpr2E* in the posterior compartment (red). Endocytosis was inhibited for 3 hours and the discs were either fixed immediately (A and D) or washed for 30 minutes (B and E) or for 60 minutes (C and F) in ice-cold cell culture media, as indicated. The distribution of Lipophorin (A-C) and of LTP (D-F) is shown in green, also in separate channels as indicated. Scale bar: 50μm.

Our results suggest that LTP recruitment to the plasma membrane mediated by a subset of lipophorin receptor isoforms is an essential component of the lipid uptake mechanism. Expression of Lpr2E but not of Lpr2F, also promotes a stabilization of lipophorin in the extracellular matrix of imaginal disc cells that is visible by an immunostaining protocol performed without permeabilization of cell membranes. This lipophorin stabilization might be related to the lipid transfer process [13]. Thus, we decided to examine whether LTP recruitment to cells was required for lipophorin stabilization or whether they were independent phenomena. To silence *apoLTP* and test for lipophorin stabilization in imaginal discs, we expressed a *UAS-apoLTPi* and a *UAS-Lpr2E* transgenes simultaneously in the fat body (*Cg-gal4*) and in imaginal discs (*hh-gal4*). Expression was temporally controlled by the Gal80^ts^ technique [24]. We reasoned that *UAS-apoLTPi* expression would silence *apoLTP* in the fat body and have no effect in imaginal discs, where *apoLTP* is not expressed. To exclude the possibility that *UAS-Lpr2E* expression in the fat body would affect lipophorin secretion, we examined lipophorin levels in the hemolymph of these larvae two days after the activation of the transgenes. We did not see a difference with the wild type, even though LTP was undetectable (Fig 5C), validating the use of these animals. As previously reported, *UAS-lpr2E* overexpression in otherwise wild type imaginal discs induced the stabilization of lipophorin in basolateral cell membranes (Fig 5A). However, this accumulation was strongly diminished in the *apoLTP* silenced animals described before (Fig 5B), in which LTP is undetectable in imaginal discs and fat body (S9 Fig). Thus, Lpr2E-mediated extracellular stabilization of lipophorin requires circulating LTP and probably, its recruitment to the plasma membrane. If this conclusion were correct, we would expect that the capacity of Lpr2E-Lpr2F chimeras to bind LTP would parallel their ability to promote lipophorin extracellular stabilization. To examine this prediction, we used the assay described in the previous section, the expression of Lpr2E, Lpr2F and their chimeras under conditions of blocked endocytosis. Overexpression of Lpr2E induced an accumulation of lipophorin mostly in basolateral membranes (Fig 6C). In contrast, Lpr2F expression had a very limited effect (Fig 6B), even though both isoforms were detected at similar levels at the plasma membrane under these conditions, as shown by extracellular immunostaining (Fig 6A). We then tested chimeras *UAS-Lpr2F+LA1*, *UAS-Lpr2F+LA1+ED*, *UAS-Lpr2F+NCN* and *UAS-Lpr2F+LA1+NCN* (Fig 6D–6G, see S7 Fig for a description of these chimeras). Adding module LA-1 to Lpr2F only slightly increased lipophorin accumulation (Fig 6D). However, addition of LA-1 plus ED or addition of the complete N-terminal region from Lpr2E, converted Lpr2F into a chimeric receptor with the same capacity as Lpr2E to mediate lipophorin accumulation (Fig 6E and 6G). The LA-1 domain was essential for this increased accumulation, since the chimera *UAS-Lpr2F+NCN* that lacks LA-1 had almost no effect (Fig 6F). Thus, the extended LA-1 domain is required both, for LTP binding (Fig 4) and for robust lipophorin accumulation in the plasma membrane (Fig 6). Together with our previous results, this strongly suggests that lipophorin receptor-mediated LTP recruitment to the plasma membrane helps stabilize lipophorin. The molecular mechanisms involved are unclear. Lipophorin receptors, LTP and lipophorin could bind cooperatively. However, in co-IP experiments Lpr2E readily pulled down LTP from hemolymph but lipophorin could not be detected above background (Fig 1A), suggesting a trimeric complex does not form in vitro. Alternatively, lipophorin stabilization could represent a functional intermediate during the process of lipid transfer to cells, maybe by direct contact between lipophorin particles and LTP. In this direction, we observed that Lpr2E-mediated lipophorin accumulation in cell membranes was transient. A 30 minutes wash of unfixed imaginal discs in ice-cold cell culture media strongly reduced lipophorin signal (Fig 7A and 7B). In contrast, LTP staining was not affected, even after a 60 minutes wash (Fig 7D–7F), further suggesting that a stable Lpr2E-LTP-lipophorin complex does not form. Rather, transient interactions between lipophorin and LTP could be limited to the duration of the lipid transfer process. Additional experiments would be required to reach a definitive conclusion.

## Discussion

During development and growth, tissues exhibit changing requirements for an external supply of lipids. For instance, oocyte maturation involves a massive uptake of neutral lipids from hemolymph. Development of *Drosophila* imaginal discs is also accompanied by an increase in intracellular lipid droplets, which is most striking in the wing pouch area of the wing discs. This accumulation is mediated, at least in part, by the expression of lipophorin receptors of the LDLR family in the area [13]. However, the molecular mechanisms involved are still unclear. It was shown that blocking endocytosis did not inhibit neutral lipid uptake [13], ruling out a mechanism similar to the uptake of cholesterol by human LDLR [27]. This conclusion is consistent with biochemical studies indicating that in insects, lipophorin functions via a reusable shuttle mechanism [28,29]. An alternative model, inspired by mammalian chylomicron and VLDL metabolism [30], posits that lipophorin binding to the lipophorin receptors bring these particles near the plasma membrane where putative lipases and lipid transporters associated to the plasma membrane or to the extracellular matrix make lipophorin lipids available to cells [2,29]. However, lipophorin is detected at high levels in the extracellular matrix of most cells by immunohistochemistry, independently of lipophorin receptors expression. This localization is mediated, at least in part, by the interaction of lipophorin with heparan sufate proteoglycans (HSPG) [31]. Thus, recruitment of lipophorin to the cell surface is not sufficient for lipid uptake. The results we present here support a different model in which the central event that initiates the transfer of lipids to imaginal disc cells and oocytes is the recruitment of the lipoprotein LTP to the plasma membrane mediated by a subset of lipophorin receptor isoforms. Three lines of evidence support this conclusion. First, lipophorin receptor isoforms that mediate lipid uptake also promote the recruitment of LTP to the plasma membrane (Figs 1, 2, S8 and [13]). Second, deletion of the LA-1 domain in the lipophorin receptors disrupts LTP binding and also impairs lipid uptake (Fig 4 and [13]), strongly suggesting that LTP binding is required for lipophorin receptor-mediated neutral lipid uptake. Third, lipophorin receptors induce a stabilization of lipophorin in the plasma membrane associated to the lipid uptake process. We showed that LTP is required for this stabilization (Figs 5 and S9). Accordingly, only the lipophorin receptor isoforms and chimeras that bind LTP and mediate lipid uptake are also able to induce lipophorin stabilization (Fig 6). Finally, we show that animals with low levels of circulating LTP display a severe reduction in the lipid content of ovaries and imaginal disc, a phenotype that is consistent with a local requirement of LTP for lipid uptake (Figs 3 and S6). However, since LTP is also required for the loading of lipophorin with lipids in the gut (S4 Fig and [4]), the decreased lipid content of lipophorin in the hemolymph of these animals could also contribute to the previous phenotype. The model we propose is consistent with the biochemical activity described for LTP in insects. In particular, experiments in which *Bombyx mori* ovarioles were cultured in medium containing radiolabeled lipophorin indicated a transfer of DAG from lipophorin to ovarioles. This transfer was inhibited by anti-LTP antibodies and restored by the addition of purified LTP, demonstrating an essential and local role of LTP in lipid uptake [18].

At the mechanistic level, LTP was suggested to use a carrier mechanism. LTP would acquire and store a limited amount of lipids from a donor lipophorin particle or cell and subsequently transfer them to a receptor. This process would not require the formation of a ternary complex between donor, acceptor and LTP [32]. Interestingly, electron microscopy studies showed that LTP particles displayed a remarkable shape with two well differentiated regions, a spherical, lipid containing head and a tail region that appeared to include a flexible hinge. It was suggested that this flexible tail would allow LTP to alternate between two conformations, contacting with lipid donor and acceptor during the lipid transfer process [15,33]. These observations prompt us to speculate that during lipid uptake, LTP recruited to the plasma membrane by the lipophorin receptors might alternately contact lipophorin particles and the plasma membrane, transferring an amount of DAG and possibly other lipids in each cycle. The interaction between LTP and lipophorin might transiently stabilize lipophorin in the extracellular matrix, as suggested by our results (Figs 5–7 and S9). Binding of the lipophorin receptors to LTP involves the LA-1 and ED domains (Fig 4). It is tempting to speculate that they would bind to LTP tail, which would leave the head region free to interact with lipophorin and the plasma membrane. An important question still completely unsolved is how lipids are incorporated into the cell. Lipids could be directly added to the lipid bilayer by LTP or alternatively, transmembrane lipid transporters might be required.

A surprising finding from our study is that all lipophorin receptor isoforms display low affinity for lipophorin, since we could not detect an interaction between Lpr2E or Lpr2F and lipophorin by co-IP, even though LTP interaction with Lpr2E was readily detected (Fig 1A). This is also supported by our in vivo results. Lpr2F overexpression promotes a very weak accumulation of lipophorin in imaginal discs under conditions of blocked endocytosis (Fig 6B). Isoform Lpr2E induces a much higher accumulation (Fig 6C) but this effect is indirect, since it requires LTP (Fig 5). More generally, we observed that lipophorin accumulation in the extracellular matrix of imaginal discs was very labile since short incubations of the unfixed tissue with ice cold buffer strongly reduced lipophorin staining (Fig 7). These observations suggest that there is a pool of lipophorin weakly associated to the extracellular matrix through low affinity interactions with HSPG [31], the lipophorin receptors or other still unidentified receptors. Entrapment of lipophorin in extracellular spaces close to the plasma membrane was described in other insects by electron microscopy [34]. This lipophorin pool would be in a dynamic equilibrium with hemolyph lipophorin, allowing lipid-depleted particles generated after the transfer of their lipid cargo to cells to quickly be replaced by lipid-rich lipophorin from the hemolymph. A high affinity binding of lipophorin to its receptors would impair such exchange.

A pending issue in our understanding of insect lipid metabolism is the role of lipophorin endocytosis [35–37]. As mentioned above, there is compelling evidence in *Drosophila* and in other insects indicating that endocytosis is not required for neutral lipid uptake. Blocking endocytosis with a *rab5* ^*2*^ allele did not hamper lipid uptake in *Drosophila* ovaries [13]. Similarly, we blocked endocytosis for 8 hours in clones of *shi* ^*ts*^ homozygous cells in imaginal discs and did not observe changes in the lipid droplets accumulation pattern (S2 Fig), suggesting that also in imaginal discs, endocytosis is not required for lipid uptake. Moreover, it was shown that chemical inhibition of the endocytic pathway did not interfere with LTP-mediated lipid exchange between lipophorin and the fat body in locust [19,35]. However, other data indicates that lipophorin particles are endocytosed in certain tissues. For example, locust fat body explants internalized fluorescently labeled lipophorin in vitro, an activity that was maximal in young adults. The internalized particles did not accumulate in the fat body and were suggested to be resecreted [35,37]. In addition, lipophorin receptors induced lipophorin endocytosis in transfected mammalian cells [12,36] or insect cells [37]. Also, overexpression of the lipophorin receptors induced lipophorin endocytosis in imaginal discs [23,38]. Interestingly, we observed that all tested lipophorin receptor isoforms induced lipophorin endocytosis in imaginal discs irrespectively of their capacity to mediate the acquisition of neutral lipids (S2 Fig). Thus, available data clearly indicates lipophorin receptors endocytic activity and their capacity to mediate cellular acquisition of neutral lipids are independent of each other. However, it is still possible that endocytosis is important for the acquisition of minor lipid species present in lipophorin [35].

LTP plays additional roles besides mediating lipophorin receptor-dependent lipid transfer from lipophorin to tissues. In particular, LTP is critical for the loading of lipophorin with lipids in the midgut (S4 Fig and [4,16,17]). However, in this case the molecular mechanisms involved appear to be different. First, in the midgut LTP is mostly found in the cytoplasm of enterocytes and not in the cell surface [4]. Second, the lipophorin receptors are not essential for LTP activity in the midgut since *lpr1* ^-^, *lpr2* ^-^ animals do not display the massive increase in gut lipids characteristic of *ApoLTP* mutants. The receptors that mediate LTP endocytosis in the midgut are not known. We have shown that Lpr2E is able to endocytose LTP in imaginal disc (Fig 1C and 1F and 1G). Since the lipophorin receptors are also expressed in the midgut [39], they could contribute to LTP endocytosis, even though other redundant receptors must necessarily exist. LTP was also shown to mediate the bidirectional transfer of lipids between the larval fat body and lipophorin in *Manduca sexta* [19]. In this direction, we observed that Lpr2E overexpression in the fat body increases LTP in the plasma membrane and also induces an LTP-dependent accumulation of lipophorin (S9 Fig), pointing to a potential role of these receptors in LTP activity in the fat body. However, in lipophorin receptor mutants LTP distribution in the fat body does not significantly change (S3 Fig), suggesting that other LTP receptors must exist.

An important question in lipid metabolism concerns the selectivity of lipid transfer between lipophorin and tissues. Our results suggest that expression of the lipophorin receptors promotes the transfer of DAG to cells, where it accumulates as TAG in lipid droplets, a process that requires LTP. However, uptake of other lipid species could also be facilitated by LTP. In this direction, LTP was shown to catalyze the transfer of DAG, phospholipids, hydrocarbons and cholesteryl esters between lipoproteins in vitro, but not of cholesterol, which can be exchanged through the aqueous phase [40]. However, the rates of facilitated transfer were found to be variable and dependent on the specific nature of the donor and acceptor particles, making it difficult to extrapolate to LTP specificity in vivo. In this direction, it was reported that a*poLTP* silencing in *Drosophila* induced changes in the composition of lipophorin DAG and sterols, suggesting LTP participates in the loading of these lipid species into lipophorin [4]. Thus, the lipophorin receptors, by recruiting LTP, may promote the uptake of most lipid species present in lipophorin. On the other hand, proteins that mediate the uptake of lipids with a high degree of specificity have also been described. In *Bombyx mori*, the CD36 proteins SCRB15 and Cameo2 mediate the selective uptake of β-carotene and lutein respectively into the silk gland despite both carotenoids being similarly transported in lipophorin particles [41]. Unfortunately, the molecular basis of this selectivity or the participation of the lipophorin receptors or LTP in the process is unknown.

## Materials and Methods

### Genetics

The following alleles and transgenes were used: *shi* ^*ts*^ [42], *Df(3R)lpr2* and *Df(3R)lpr1/2* [13], *UAS-Rab5-GFP* [43], *UAS-Lamp-GFP* [44], *UAS-apoLpp-RNAi* (stock 106311 from VDRC), *hh-Gal4* [45], *Cg-Gal4* [25], *FB-Gal4* [46], *V32-Gal4* (a gift from Daniel St Johnston) and *tub-Gal80* ^*ts*^ [24].

To generate the *apoLTP[excDG06206]* mutation, we induced the imprecise excision of transposon *P{wHy}DG06206* following the protocol described in [47]. The extent of the deletion was mapped by inverse PCR and sequencing of the resulting fragments.

Clones of cells homozygous for the *shi* ^*ts*^ allele in imaginal discs were induced by heat shocking *shi* ^*ts*^,*FRT9-2/Ubi-GFP*,*FRT9-2;hs-flp/+* larvae for one hour at 37°C, 48–72 hours AEL. After heat shock, larvae were cultured at 18°C and switched to the restrictive temperature (33°C) for 8 hours before dissection. Blocking endocytosis for 10 hours or more induced severe morphogenetic phenotypes in imaginal discs.

To overexpress Lpr2E in the posterior compartment of wing imaginal discs in animals with reduced levels of LTP, the following genotype was used: *Cg-gal4*, *tub-gal80* ^*ts*^/*UAS-lpr2E*;*hh-gal4*/*UAS-apoLTP-RNAi*. The cross was maintained at 18°C and mid third instar larvae were transferred to 29°C for two days to activate the UAS transgenes. Control larvae shown in Fig 6 did not carry the *Cg-gal4*, *tub-gal80* ^*ts*^ chromosome. Additional controls were performed with larvae of genotype: *Cg-gal4*,*tub-gal80* ^*ts*^/*UAS-lpr2E*;*hh-gal4*/*+*, similarly obtaining a robust lipophorin stabilization in the posterior compartment.

To examine protein distribution under conditions of blocked endocytosis, male larvae of the following genotype: *shi* ^*ts*^/Y;*UAS-lpr2X*/+;*hh-Gal4*/+, where *UAS-lpr2X* stands for the different chimeras tested, were placed inside a glass tube and submerged in water at 33°C for 2.5 or 3 hours. Afterwards, they were immediately transferred to ice and dissected at 4°C.

### Transgenes and molecular biology

To generate *UAS-lpr2E_ecto_TAP*, a *lpr2E* cDNA fragment coding from Met1 to Glu985 was flanked by Kpn I and Xba I restriction sites and cloned in frame into pUAST-CTAP [48]. *UAS-lpr2F_ecto_TAP* was similarly generated using a fragment comprising from Lpr2F Met1 to Glu782. In both constructs, the transmembrane and intracellular domains were deleted, generating secretable proteins fused to a C-terminal TAP tag. To generate plasmids for expression of full length Lpr2E and Lpr2F proteins fused to a C-terminal TAP tag in the germ line, we first cloned the tag CTAP flanked by engineered Xba I and Spe I sites into the Xba I site of pUASp [49], creating pUASp_TAP. Full-length lpr2E or lpr2F DNA fragments excluding the stop codons and with engineered Kpn I and Xba I flanking sites were cloned in frame into pUASp_TAP, generating UASp-lpr2E_TAP and UASp-lpr2F_TAP. Transgenic flies were generated with all four plasmids.

The following transgenes to overexpress Lpr2E, Lpr2F and chimeras as a fusion to a C-terminal 3xHA tag were described in [13]: *UAS-lpr2E*, *UAS-lpr2F*, *UAS-lpr2F+LA1+NCN*, *UAS-lpr2F+LA1*, *UAS-lpr2F+NCN*. To generate *UAS-lpr2+LA1+ED*, first a DNA fragment coding for ED+LA1 domains of Lpr2E (from Leu170 to Thr232) was flanked by Not I sites (each coding for 3 Ala) and cloned into pAc-lpr2F-NotI [13] and transferred to pUASTattb [50]. To introduce a Myc tag into Lpr2E and Lpr2F extracellular domains, NotI sites were first engineered after Lpr2E Glu920 or after Lpr2F Glu717, located between the EGF-C module and the O-glycosylation region. Not I flanked fragments containing six copies of a Myc tag were generated by PCR using pCS2+NLS MT vector as template [51] and cloned into the engineered Not I sites. These cDNAs, also containing a C-terminal 3xHA tag, were transferred to pUASTattB to generate *UAS-lpr2E-Myc* and *UAS-lpr2F-Myc*.

To silence *ApoLTP* by RNA interference (RNAi), an 874 base pair genomic fragment corresponding to part of *ApoLTP* 7^th^ exon was cloned as an inverted tandem repeat containing an intervening 81 base pair region into pBluescript (Stratagene). The tandem repeat was transferred to pUAST and transgenic flies were obtained (*UAS-ApoLTPi*).

To generate an *ApoLTP-myc* genomic rescue transgene, we started from the attB-P[acman]-Cm^R^-BW based genomic clone CH321-38C23 [52], which contains a 84241 base pair fragment that includes *ApoLTP*. A 6XMyc tag was inserted in *ApoLTP* C-terminus (after Ser4333) and a V5 tag was inserted after Arg23, two amino acids after the predicted signal peptide cleavage site, by recombineering [53]. We could not obtain transgenic flies with this modified BAC clone, possibly because of its length. Thus, we deleted sequences downstream of *ApoLTP* by recombineering, generating a BAC clone containing a 43241 base pair insert which includes 25645 base pairs upstream and 5087 base pairs downstream of *ApoLTP* CDS. With this shorter BAC, we generated transgenic flies at the CBMSO transgenesis facility. This genomic clone, even though it rescues a null *ApoLTP* mutant, is expressed at lower levels than the endogenous gene. For unknown reasons, we cannot detect the protein using α-V5.

### Affinity purification

*UAS-lpr2E_ecto_TAP* and *UAS-lpr2F_ecto_TAP* were expressed in the larval fat body using the driver *FB-gal4*. Notice that *UAS-lpr2E_ecto_TAP* overexpression delayed growth but eventually larvae reached normal size. Wandering 3^th^ instar larvae were collected, washed and frozen in liquid nitrogen. 6 g per genotype were used in the experiment. Larvae were powdered in a mortar and pestle in liquid nitrogen and the powder was added to 50 ml of ice cold extraction buffer (20 mM K-HEPES pH 7.9, 50 mM KCl, 100 mM NaCl, 2 mM DTT, 0.5 mM CaCl2, 0.5 mM PMSF, 1x protease inhibitor cocktail from Roche). The extract was centrifuged at 4000 rpm in a falcon tube for 5 minutes and the supernatant filtered successively through a 2.7 μm and 0.7 μm syringe filters fitted with glass microfiber pre-filters to reduce clogging. For the affinity purification step, we essentially followed the protocol from Puig et al. [22] with some modifications. In particular, the cleared lysate was incubated for 2 hours with 500 μl of IgG-sepharose matrix (GE) previously equilibrated with extraction buffer. Beads were then washed five times in IPP150-Ca buffer (10 mM Tris-HCl pH8.0, 150 mM NaCl, 0.5 mM CaCl2, 0.1% triton X100) and resuspended in CBB buffer (10 mM Tris-HCl pH 8, 150 mM NaCl, 0.1% triton X100, 2 mM CaCl2, 10 mM β-mercaptoethanol, 1 mM magnesium acetate, 1 mM imidazole). 30 μl of TEV protease (10u/μl) were added and the reaction allowed to proceed over night at 4°C. The supernatant and two additional 500 μl washes in CBB buffer were pooled and incubated with 500 μl of calmodulin-sepharose matrix (GE) for 4 hours at 4°C. Beads were then washed six times for a total time of 30 minutes in CBB buffer and eluted in 500 μl of CEB buffer (10mM Tris-HCl pH8.0, 10 mM β-mercaptoethanol, 150 mM NaCl, 1 mM magnesium acetate, 1 mM imidazole, 0.1% triton X-100, 20 mM EGTA) for 20 minutes at 4°C. Proteins were precipitated from the supernatant using the 2-D Clean-Up kit (GE), resuspended in Laemmli buffer and separated by PAGE. Proteins were stained with colloidal Coomassie and bands differentially present in the samples were excised and identified by peptide mass fingerprinting and peptide fragmentation at the "Parque Científico de Madrid" facility.

*UASp-lpr2E_TAP* and *UASp-lpr2F_TAP* were expressed in the germ line driven by *V32-gal4*. 250 ovaries from 4–5 days old females fed with yeast paste were dissected and homogenated in a Tenbroeck tissue grinder in 500 μl ice cold lysis buffer (10mM Tris-HCl pH 7.6, 150 mM NaCl, 0.5 mM CaCl2, 0.1% Triton X-100, 0.5 mM PMSF and 1x Roche protease inhibitor cocktail). The homogenate was kept on ice for 10 minutes and centrifuged at 16,000g for 15 minutes. Cleared lysates from 4,000 ovaries were pooled and filtered through a 0,22 μm syringe filter, obtaining a total of 5.5 ml of extract. Affinity purification of Lpr2E_TAP and Lpr2F_TAP was performed in a single step using IgG coated dynabeads. Conjugation of dynabeads M-270 Epoxy (Invitrogen) with rabbit IgG was performed as described [54]. 5.5 ml of ovary extract was incubated with 30 mg IgG-coated dynabeads for 2 hours at 4°C with continuous shaking. The magnetic beads were then washed five times in cold lysis buffer for a total of 30 minutes and bound proteins eluted by cleavage of the TAP tag with TEV protease (150 u) in 200μl of lysis buffer supplemented with 1 mM DTT during 3 hours at 4°C. Proteins were precipitated from the supernatant, separated and processed for mass spectrometry analysis as before.

### Co-immunoprecipitation

To express Lpr2E-HA and Lpr2F-HA in S2 cells, UAS-lpr2E or UAS-lpr2F [13] plasmids were co-transfected with pAC-Gal4 [55]. About 4.5 million cells were lysated in 300 μl lysis buffer (150mM NaCl, 20 mM Tris HCl pH 7.8, 0.5% triton X-100, 1 mM DTT, 0.5 mM PMSF, 1x Roche protease inhibitor cocktail) by one cycle of freezing and thawing. The lysate was cleared by centrifugation at 16,000 g for 5 minutes and added to protein G dynabeads (Invitrogen) conjugated to mouse anti-HA (Santa Cruz Biotechnology) following manufacturer instructions. Beads were incubated with the lysate for 10 minutes at room temperature, washed twice with lysis buffer and incubated with 100 μl of diluted hemolymph for one hour at 4°C with shaking. After three washes in washing buffer (150 mM NaCl, 20 mM Tris-HCl pH 7.8, 0.1% triton X-100), proteins were eluted from dynabeads by boiling in Laemmli buffer for 4 minutes. Diluted hemolymph was prepared as follows: 50 wild type wandering larvae were washed, dried and placed in 350 μl of ice cold hemolymph buffer (150 mM NaCl, 20 mM Tris-HCl pH7.8, 1 mM DTT, 0.5 mM PMSF, 1x Roche protease inhibitor cocktail). Under the dissecting microscope, larvae were pierced with a pair of forceps so that hemolymph bled out into the buffer. The diluted hemolymph was collected and centrifuged for 5 minutes at 5,000 rpm to remove cells and tissue debris. Diluted hemolymph was used immediately after being prepared.

### Immunohistochemistry

The following antibodies were used in this work: rabbit α-ApoLTPI and α-ApoLTP II [4], rabbit α-LpFL and rabbit α-LpII [5], rat α-HA (Roche), mouse α-HA (Santa Cruz Biotechnology) and mouse α-Myc (DSHB). Immunostaining of imaginal discs and ovaries as well as immunostaining of extracellular proteins was performed as described [13]. To examine the stability of lipophorin association to Lpr2E in vivo (Fig 7), wing imaginal discs were dissected in Sf-900 II SFM culture media (gibco) at 4°C and incubated in the same media for 30 minutes or 60 minutes, also at 4°C. They were subsequently fixed and processed following standard protocols. Lipids were visualized by Nile red or by oil red O stains. For Nile red, fixed imaginal discs or ovaries were incubated with 0.002% Nile red dye diluted in PBS and 0.3% triton X-100 for 60 minutes and washed for 10 minutes in the same buffer without the dye. For oil red O stain, fixed imaginal discs were incubated in a 0.5% solution of oil red O in propylene glycol at 60°C for one hour and then washed twice in 85% propylene glycol and three times in PBS, essentially as described in [56].

### Hemolymph extraction

To quantitatively compare LTP and lipophorin levels in hemolymph, we placed two washed and dry larvae on a piece of parafilm on ice and pierced them with a pair of forceps. Hemolymph was collected by capillarity filling 0.5 μl glass micropipettes (Drummond) and immediately transferring the contents to 20 μl Laemmli buffer for western blot analysis.

## Supporting Information

S1 Fig (A) Coomassie-stained SDS-PAGE gel showing pull-downs from ovary extracts of a wild type (wt) stock or from flies expressing full-length lipophorin receptors *UASp-Lpr2F_TAP* or *UASp-Lpr2E_TAP* in the germ-line driven by *V32-gal4*. TAP-tagged proteins were pulled-down in a single step using IgG-conjugated Dynabeads. (B) Similar Coomassie-stained gel showing pull-downs from total larvae extracts. The secretable *UAS-lpr2F_ecto_TAP* and *UAS-lpr2E_ecto_TAP* were expressed in the fat body driven by *FB-gal4*. The purification was carried out in two steps, using IgG-Sepharose and Calmodulin-Sepharose matrices. The following protein bands were identified by mass spectrometry: 1- ApoLTP, 2- CG8507 (α-2-macroglobulin receptor-associated protein) and 3- Peroxidasin. Asterisks indicate overexpressed TAP-tagged baits. IgG light chain position is marked by an arrow. St: molecular weight standard.
(TIF)

S2 Fig Lipophorin receptor-mediated lipophorin endocytosis in imaginal discs. (A-C) *UAS-Rab5-GFP*, an early endosome marker, and either *UAS-Lpr2E* (A and B) or *UAS-Lpr2F* (C) isoforms were expressed in the posterior compartment of wing imaginal discs driven by *en-gal4*. The lipophorin receptors, detected with an anti-HA antibody, are shown in blue, Rab5-GFP in green and lipophorin in red. Individual channels are also included for clarity. A confocal cross-section (A) and apical sections (B and C) are shown. Lpr2E and Lpr2F induce lipophorin endocytosis. Multiple vesicles containing lipophorin, Rab5 and the lipophorin receptor can be identified; some of them are indicated by yellow arrows. (D) Imaginal disc containing clones of *shi* ^*ts*^ homozygous cells. After clone induction, larvae were maintained at the permissive temperature and switched to 33°C for 8 hours before dissection to block endocytosis. Clones were identified by the absence of the marker GFP (red) and are outlined with a dashed line. Neutral lipids were imaged by Nile red staining (green), also shown in a separate panel. No changes in the pattern of lipid accumulation can be detected. Scale bars: 10μm (A-F) and 100 μm in D.
(TIF)

S3 Fig LTP distribution in larval tissues. (A-E) Tissues from wild type (first column) and *Df(3R)lpr1/2* homozygous larvae (second column) were dissected and LTP distribution analyzed (green). LTP accumulates in the fat body (A), oenocytes (B), salivary gland imaginal rings, indicated by an arrow in (C) and shown at higher magnification in the inset, gastric caeca (D) and ring gland (E). Nuclei were labeled with DAPI (blue, A-E) and F-actin with phalloidin (red, C-E). In the lipophorin receptors deficiency, LTP accumulates at slightly reduced levels in the tissues examined except for the salivary gland imaginal rings, for which no difference can be observed. Scale bars: 50μm.
(TIF)

S4 Fig Characterization of the novel allele *apoLTP[excDG06206]*. (A) Genomic map of *apoLTP* gene, with exons shown as rectangles. Coding sequences are in black and the UTR in grey. The position of the transposable element *P{wHy}DG06206* is indicated, as well as the region deleted after its mobilization (grey bar), which generated the allele *apoLTP[excDG06206]*. (B-C) Animals mutant for the null allele *apoLTP[excDG06206]* show delayed growth. (B) Staged larvae of 24–48 hours after egg laying (first instar), 48–72 hours (second instar), 72–96 hours (third instar) and 96–120 hours (white pupae) are shown. Homozygous *apoLTP[excDG06206]* larvae of the same age are displayed in (C). Growth is delayed and mutant larvae do not molt, remaining in the first instar and dying after about 10 days. The gut of wild type and *apoLTP[excDG06206]* first instar larvae are also shown to illustrate a strong accumulation of neutral lipids (yellow) in a region of the midgut (bracket) in mutant animals, as revealed by Nile red staining. Nuclei are labeled with DAPI in blue. gc: gastric caeca, mt: Malpighian tubules, fb: fat body.
(TIF)

S5 Fig Cg-gal4 expression pattern. *UAS-GFP* expression driven by *Cg-gal4* in one week old females (A, B and D) and in a third instar larva (C) is shown. GFP fluorescents (green) was imaged in live animals (A-C) and fixed tissue (D). GFP was detected in larval and adult fat body. Arrows indicate the head fat body (B) and larval fat body (C). (D) Part of an adult abdomen (bracket in A) was dissected to reveal the fat body associated to the body wall. Nuclei were labeled with DAPI (blue) and F-actin with phalloidin (red). GFP (green) is only expressed in fat body cells. Scale bar: 200 μm.
(TIF)

S6 Fig LTP is required for neutral lipids accumulation in imaginal discs. Wing imaginal discs displaying neutral lipids in red, revealed by oil red O staining. (A) Wild type. (B) *apoLTP* was silenced in the fat body by the expression of a *UAS-apoLTPi* transgene driven by *Cg-gal4* for 4 days. Since *apoLTP* silencing delays larval growth, to compare animals of equivalent developmental stages we selected white pupae, an easily recognizable stage that lasts for about one hour. Scale bar: 100μm.
(TIF)

S7 Fig Lpr2E protein domains involved in LTP binding. (A) Alignment of the N-terminal region of six lipophorin receptor proteins from *D*. *melanogaster*, *D*. *virilis*, *Musca domestica* and *Ceratitis capitata* dipteran species. Signal peptides are not included in the alignment. The LA-1 domain, highlighted by a green bar, is highly conserved. It contains six cysteines, marked with asterisks, which form characteristic disulphide bonds. Conservation extends for 16 additional amino acids N-terminally, a region we named extension domain (ED, red bar). (B) Scheme of the modular composition of isoforms Lpr2E, Lpr2F and chimeric receptors obtained by domain swapping and used throughout this work.
(TIF)

S8 Fig Wing imaginal discs overexpressing *UAS-lpr1H*, *UAS-lpr1J* or *UAS-lpr1M* isoforms in the posterior compartment as indicated, driven by *hh-gal4*. Larvae were hemizygous for a *shibire* ^*ts*^ allele and endocytosis was blocked for 3 hours prior dissection and fixation. LTP only binds *lpr1* isoforms containing an extended LA-1 domain (Lpr1H and Lpr1J).
(TIF)

S9 Fig (A-F) Larval fat body of wild type (wt, A and B), *Cg-gal4*, *tub-gal80* ^*ts*^/*UAS-Lpr2E* (C and D) and *Cg-gal4*,*tub-gal80* ^*ts*^/*UAS-Lpr2E*;*hh-gal4*/*UAS-apoLTPi* (E and F) genotypes. Larva were grown at 18°C and transferred to 29°C for two days prior to dissection to activate the UAS transgenes. LTP and lipophorin distribution are shown in red, as indicated. Nuclei are labeled with DAPI in blue and overexpressed Lpr2E-HA is shown in green, as indicated. LTP accumulates in the fat body cell membranes in the wild type (A). This accumulation is strongly potentiated by the expression of *UAS-Lpr2E* (C) and is undetectable when a *UAS-apoLTPi* transgene is co-expressed together with *UAS-Lpr2E* (E). Lipophorin can be detected in the plasma membrane and in the cytoplasm of wild type fat body cells (B). Expression of *UAS-Lpr2E* increases lipophorin accumulation in the plasma membrane (D). However, when *UAS-apoLTPi* is co-expressed together with *UAS-Lpr2E*, levels of lipophorin in the plasma membrane are similar to the wild type (F), indicating that the increased accumulation of lipophorin induced by Lpr2E requires LTP. (G-H) Imaginal discs of the indicated genotypes shown as controls for Fig 5. *UAS-lpr2E* and *UAS-apoLTPi* were expressed in the posterior compartment driven by *hh-gal4* for two days. LTP accumulates in the posterior compartment (G). However, additional expression of *UAS-LTPi* in the fat body driven by *Cg-gal4* abolishes LTP accumulation in imaginal discs (H). Note that *UAS-Lpr2E* is also expressed in the fat body in this genetic combination. Scale bar: 100μm.
(TIF)
