# Supplementary figures and images for: Drosophila Lipophorin Receptors Recruit the Lipoprotein LTP to the Plasma Membrane to Mediate Lipid Uptake
Source: PLoS Genet. 2015 Jun 29;11(6):e1005356. doi: 10.1371/journal.pgen.1005356 (PMC4486166; doi:10.1371/journal.pgen.1005356)

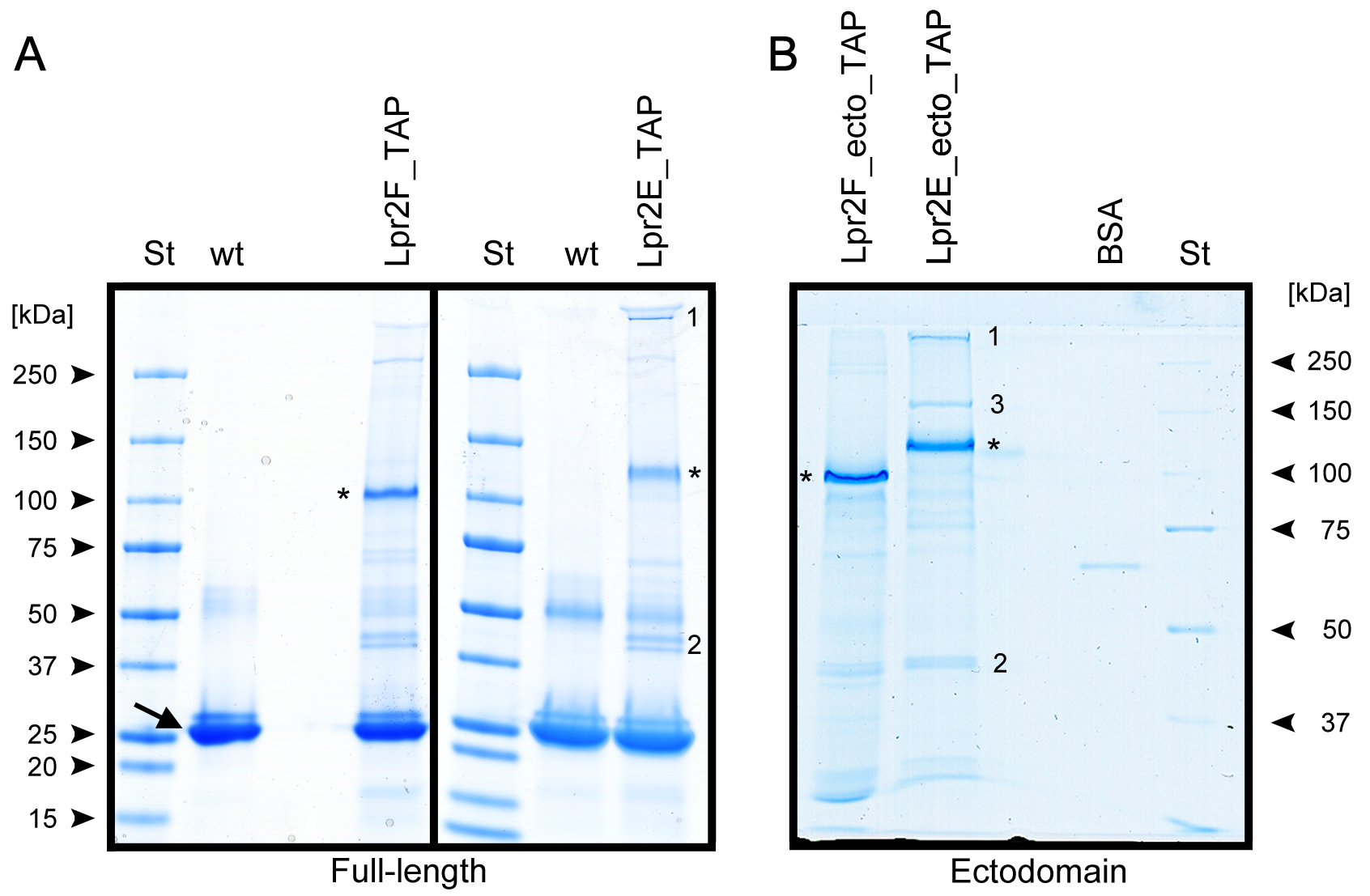

Supplement: S1 Fig — TAP-tagged proteins were pulled-down in a single step using IgG-conjugated Dynabeads. (B) Similar Coomassie-stained gel showing pull-downs from total larvae extracts. The secretable UAS-lpr2F_ecto_TAP and UAS-lpr2E_ecto_TAP were expressed in the fat body driven by FB-gal4. The purification was carried out in two steps, using IgG-Sepharose and Calmodulin-Sepharose matrices. The following protein bands were identified by mass spectrometry: 1- ApoLTP, 2- CG8507 (α-2-macroglobulin receptor-associated protein) and 3- Peroxidasin. Asterisks indicate overexpressed TAP-tagged baits. IgG light chain position is marked by an arrow. St: molecular weight standard. (TIF) [file pgen.1005356.s001.tif]

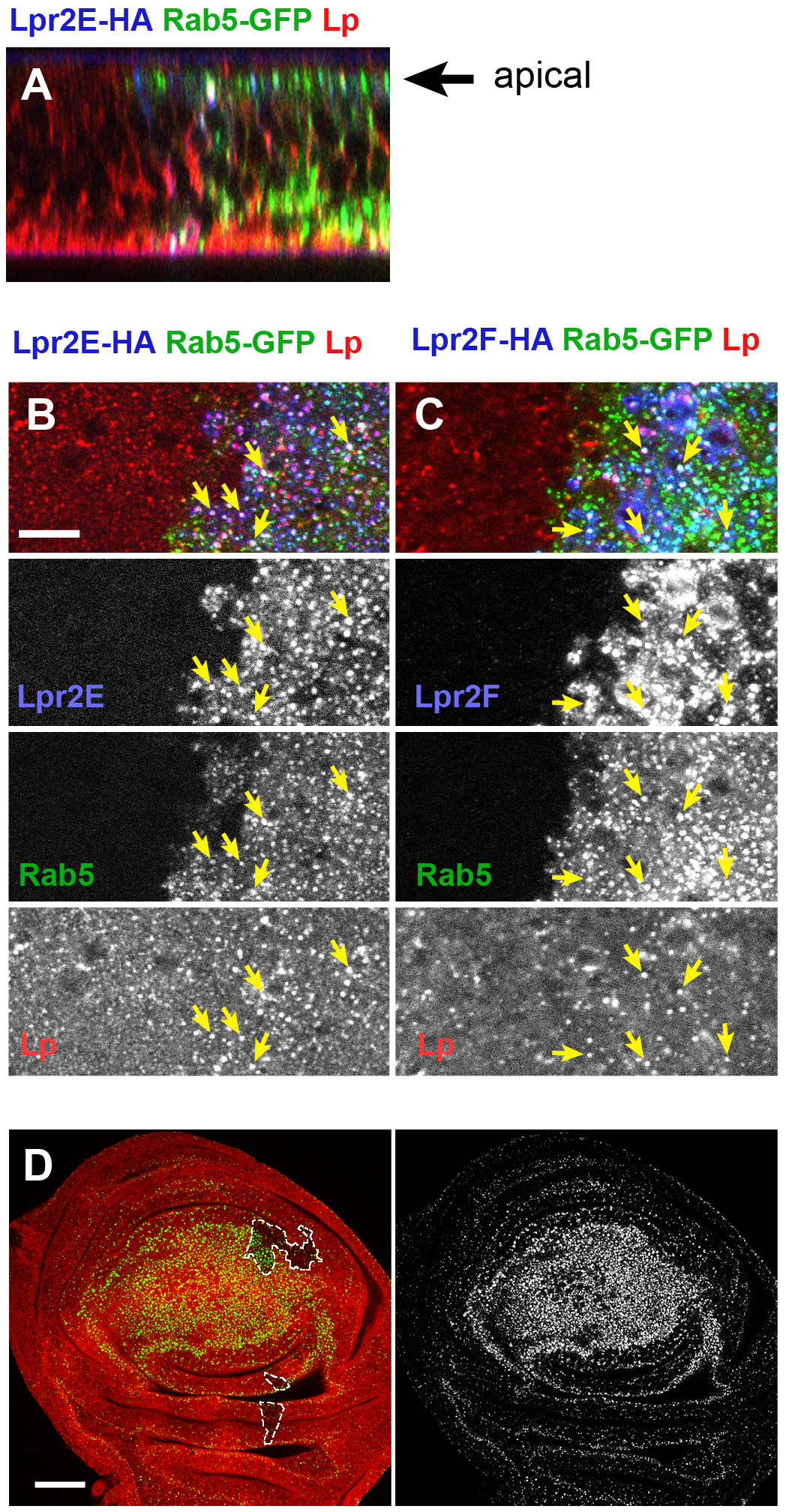

Supplement: S2 Fig — (A-C) UAS-Rab5-GFP, an early endosome marker, and either UAS-Lpr2E (A and B) or UAS-Lpr2F (C) isoforms were expressed in the posterior compartment of wing imaginal discs driven by en-gal4. The lipophorin receptors, detected with an anti-HA antibody, are shown in blue, Rab5-GFP in green and lipophorin in red. Individual channels are also included for clarity. A confocal cross-section (A) and apical sections (B and C) are shown. Lpr2E and Lpr2F induce lipophorin endocytosis. Multiple vesicles containing lipophorin, Rab5 and the lipophorin receptor can be identified; some of them are indicated by yellow arrows. (D) Imaginal disc containing clones of shi ts homozygous cells. After clone induction, larvae were maintained at the permissive temperature and switched to 33°C for 8 hours before dissection to block endocytosis. Clones were identified by the absence of the marker GFP (red) and are outlined with a dashed line. Neutral lipids were imaged by Nile red staining (green), also shown in a separate panel. No changes in the pattern of lipid accumulation can be detected. Scale bars: 10μm (A-F) and 100 μm in D. (TIF) [file pgen.1005356.s002.tif]

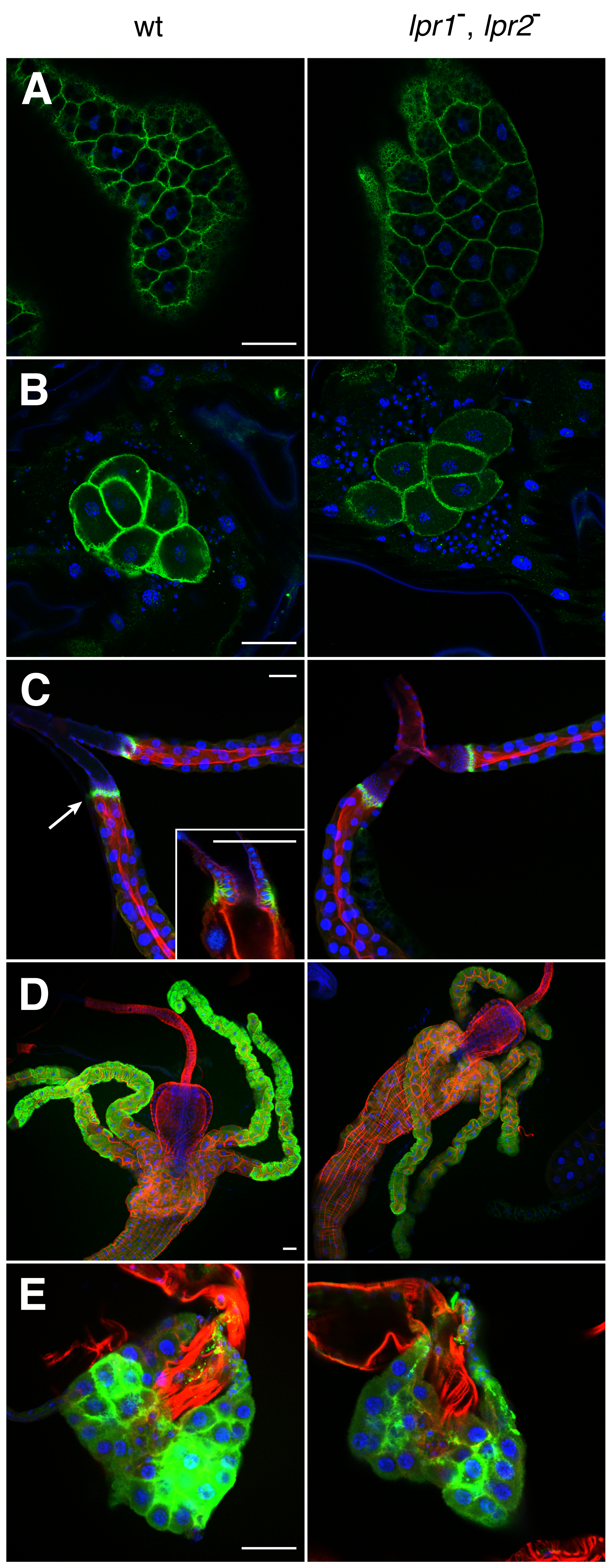

Supplement: S3 Fig — (A-E) Tissues from wild type (first column) and Df(3R)lpr1/2 homozygous larvae (second column) were dissected and LTP distribution analyzed (green). LTP accumulates in the fat body (A), oenocytes (B), salivary gland imaginal rings, indicated by an arrow in (C) and shown at higher magnification in the inset, gastric caeca (D) and ring gland (E). Nuclei were labeled with DAPI (blue, A-E) and F-actin with phalloidin (red, C-E). In the lipophorin receptors deficiency, LTP accumulates at slightly reduced levels in the tissues examined except for the salivary gland imaginal rings, for which no difference can be observed. Scale bars: 50μm. (TIF) [file pgen.1005356.s003.tif]

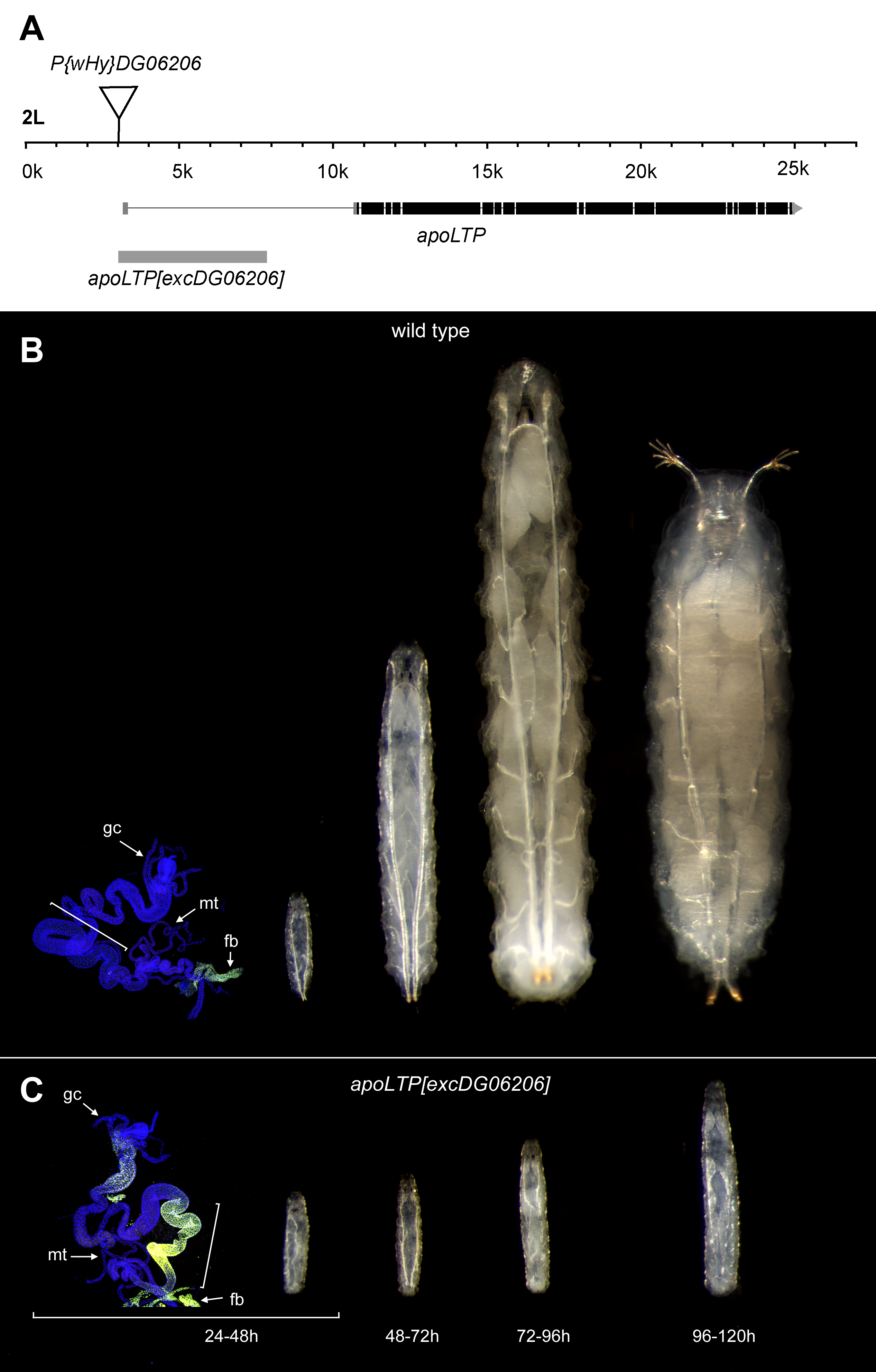

Supplement: S4 Fig — (A) Genomic map of apoLTP gene, with exons shown as rectangles. Coding sequences are in black and the UTR in grey. The position of the transposable element P{wHy}DG06206 is indicated, as well as the region deleted after its mobilization (grey bar), which generated the allele apoLTP[excDG06206]. (B-C) Animals mutant for the null allele apoLTP[excDG06206] show delayed growth. (B) Staged larvae of 24–48 hours after egg laying (first instar), 48–72 hours (second instar), 72–96 hours (third instar) and 96–120 hours (white pupae) are shown. Homozygous apoLTP[excDG06206] larvae of the same age are displayed in (C). Growth is delayed and mutant larvae do not molt, remaining in the first instar and dying after about 10 days. The gut of wild type and apoLTP[excDG06206] first instar larvae are also shown to illustrate a strong accumulation of neutral lipids (yellow) in a region of the midgut (bracket) in mutant animals, as revealed by Nile red staining. Nuclei are labeled with DAPI in blue. gc: gastric caeca, mt: Malpighian tubules, fb: fat body. (TIF) [file pgen.1005356.s004.tif]

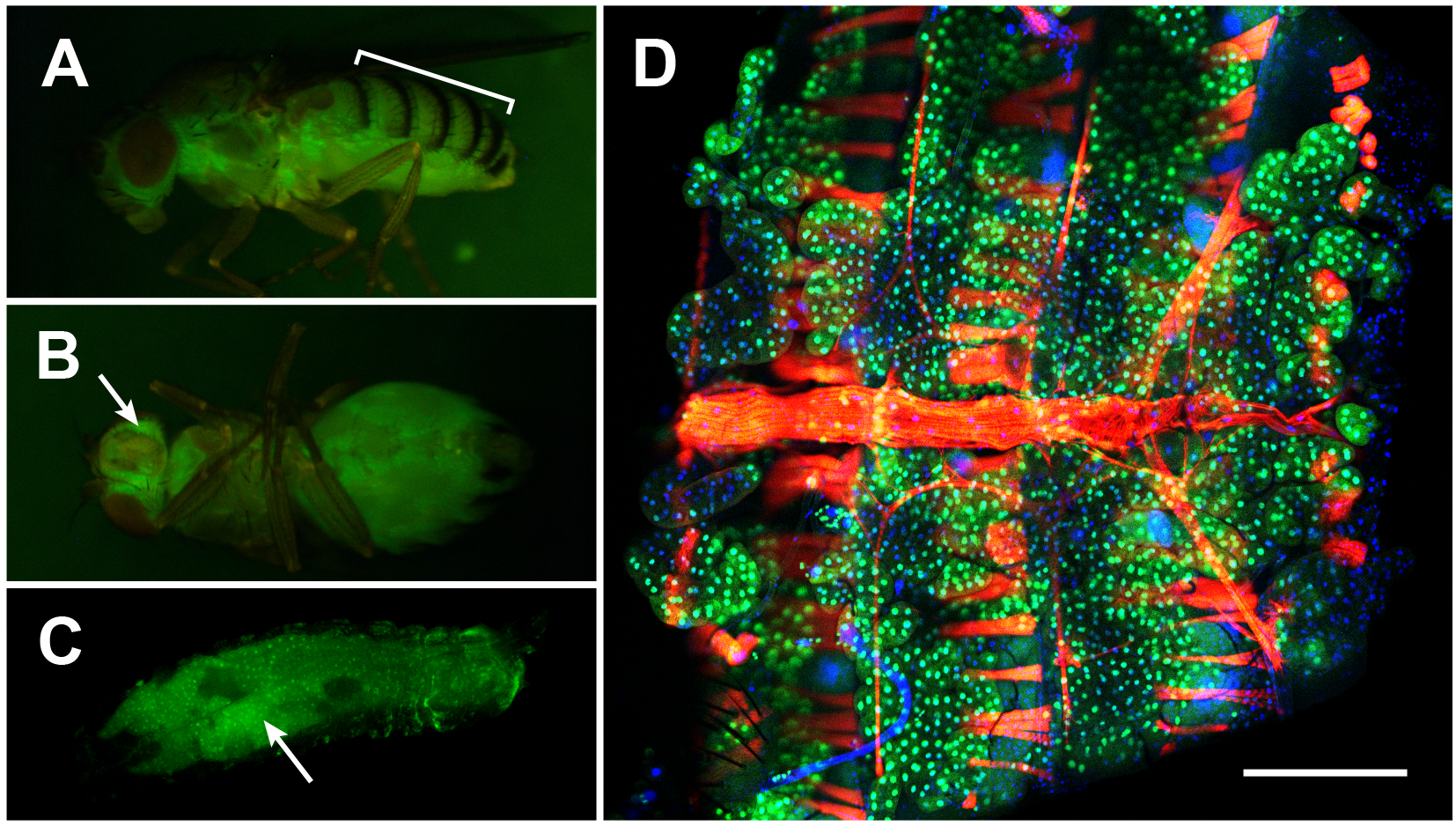

Supplement: S5 Fig — UAS-GFP expression driven by Cg-gal4 in one week old females (A, B and D) and in a third instar larva (C) is shown. GFP fluorescents (green) was imaged in live animals (A-C) and fixed tissue (D). GFP was detected in larval and adult fat body. Arrows indicate the head fat body (B) and larval fat body (C). (D) Part of an adult abdomen (bracket in A) was dissected to reveal the fat body associated to the body wall. Nuclei were labeled with DAPI (blue) and F-actin with phalloidin (red). GFP (green) is only expressed in fat body cells. Scale bar: 200 μm. (TIF) [file pgen.1005356.s005.tif]

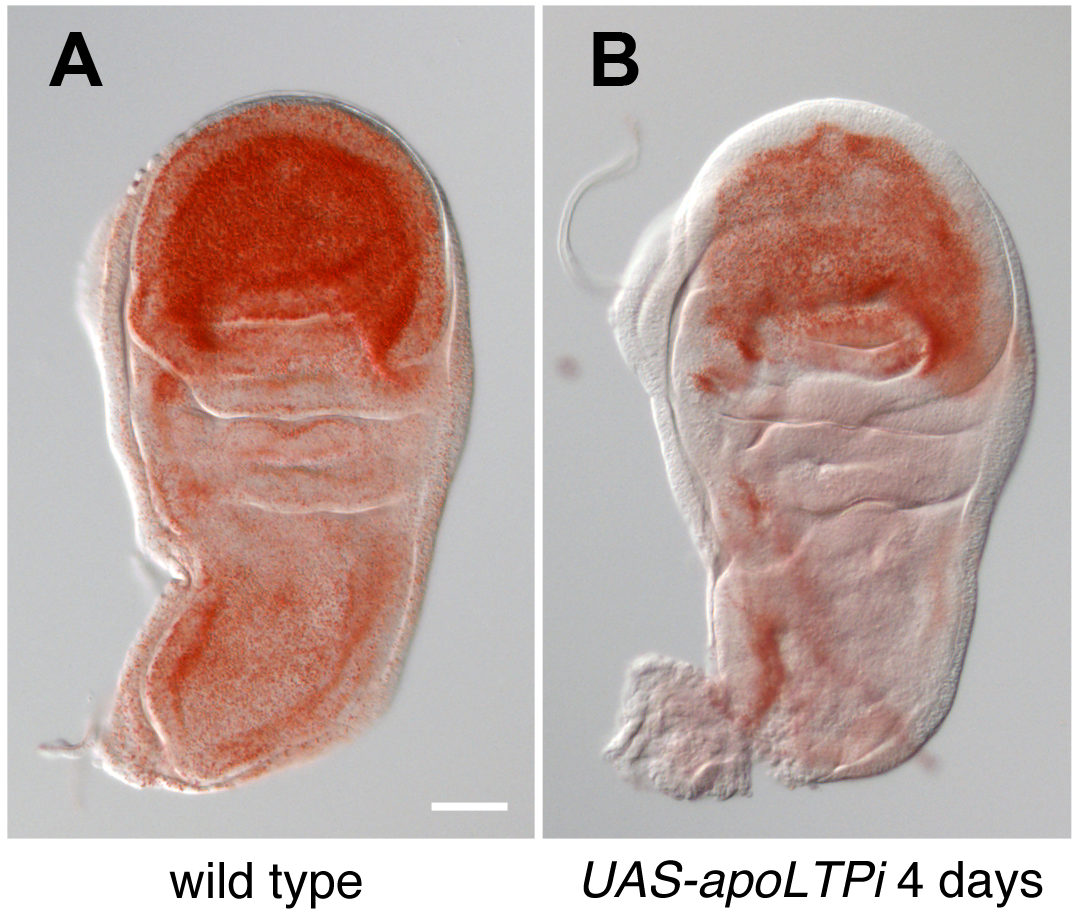

Supplement: S6 Fig — Wing imaginal discs displaying neutral lipids in red, revealed by oil red O staining. (A) Wild type. (B) apoLTP was silenced in the fat body by the expression of a UAS-apoLTPi transgene driven by Cg-gal4 for 4 days. Since apoLTP silencing delays larval growth, to compare animals of equivalent developmental stages we selected white pupae, an easily recognizable stage that lasts for about one hour. Scale bar: 100μm. (TIF) [file pgen.1005356.s006.tif]

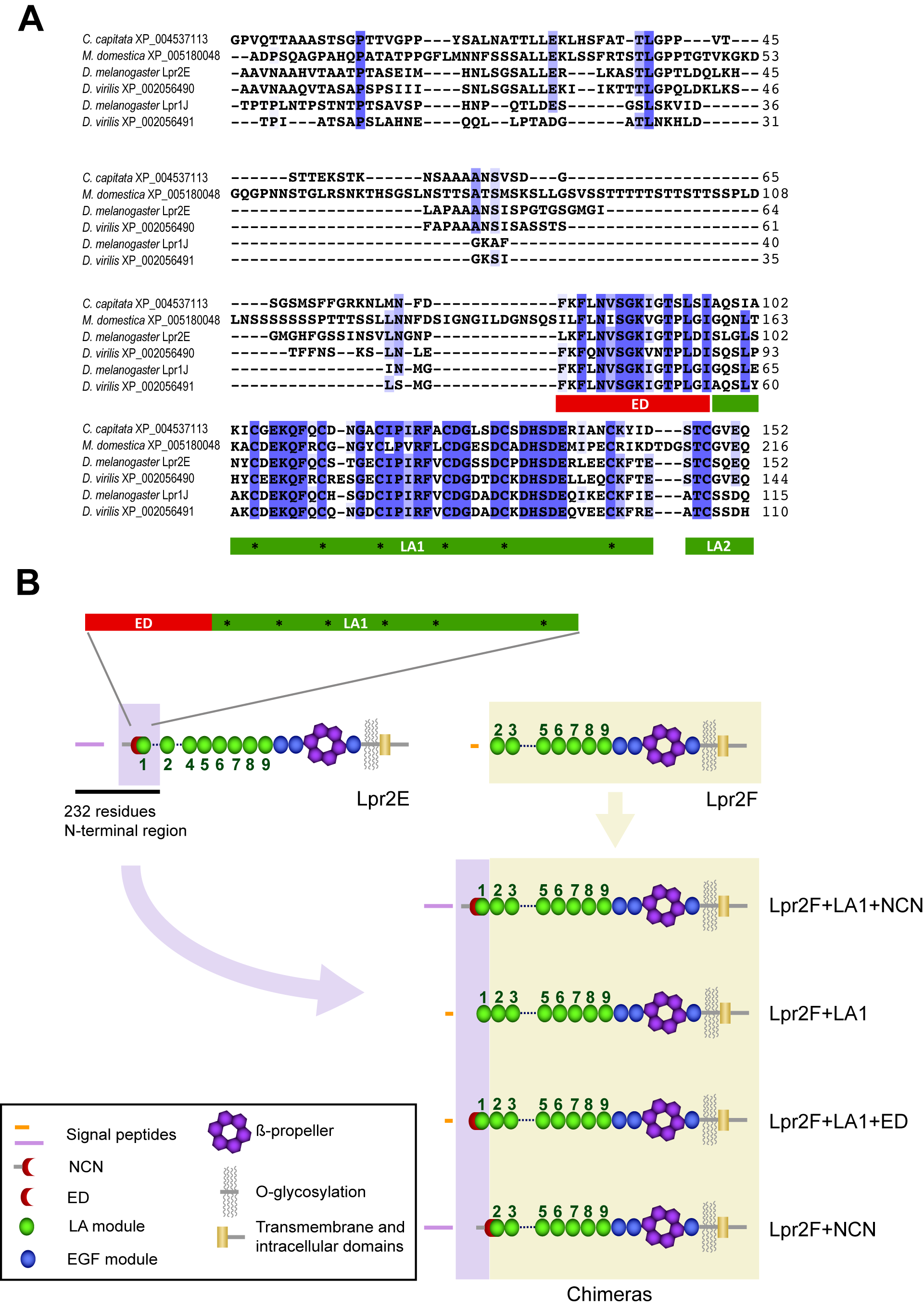

Supplement: S7 Fig — (A) Alignment of the N-terminal region of six lipophorin receptor proteins from D. melanogaster, D. virilis, Musca domestica and Ceratitis capitata dipteran species. Signal peptides are not included in the alignment. The LA-1 domain, highlighted by a green bar, is highly conserved. It contains six cysteines, marked with asterisks, which form characteristic disulphide bonds. Conservation extends for 16 additional amino acids N-terminally, a region we named extension domain (ED, red bar). (B) Scheme of the modular composition of isoforms Lpr2E, Lpr2F and chimeric receptors obtained by domain swapping and used throughout this work. (TIF) [file pgen.1005356.s007.tif]

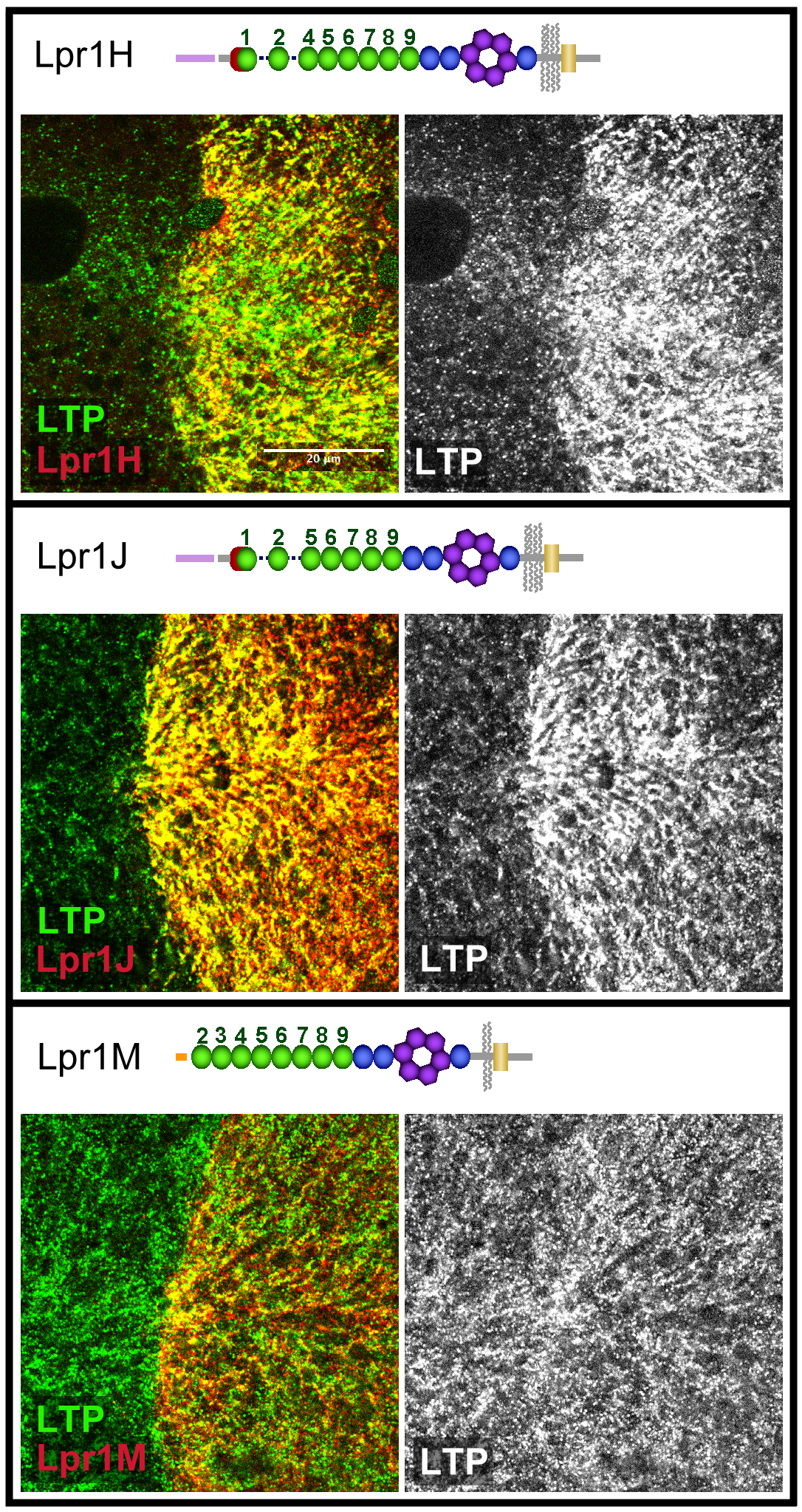

Supplement: S8 Fig — Larvae were hemizygous for a shibire ts allele and endocytosis was blocked for 3 hours prior dissection and fixation. LTP only binds lpr1 isoforms containing an extended LA-1 domain (Lpr1H and Lpr1J). (TIF) [file pgen.1005356.s008.tif]

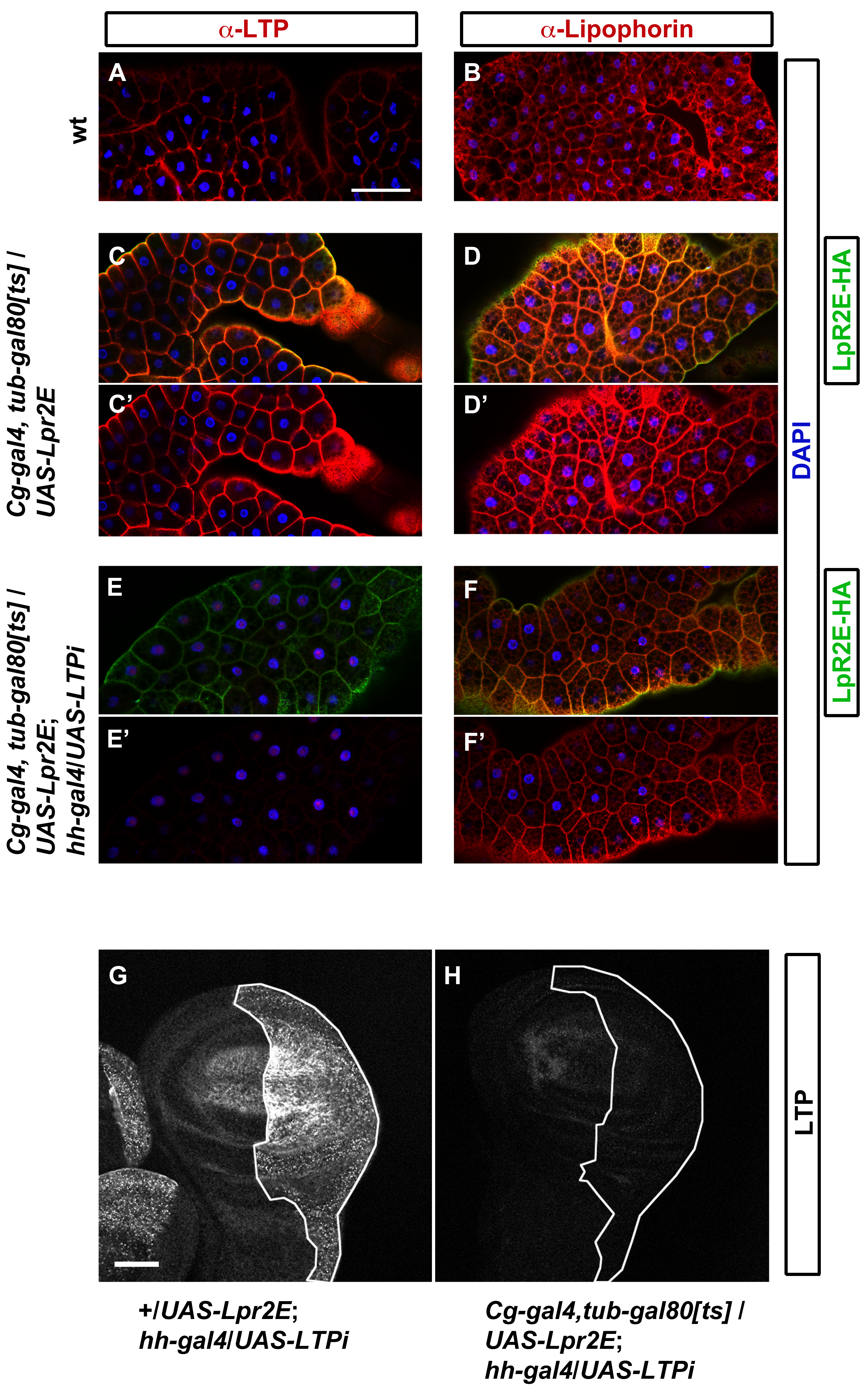

Supplement: S9 Fig — Larva were grown at 18°C and transferred to 29°C for two days prior to dissection to activate the UAS transgenes. LTP and lipophorin distribution are shown in red, as indicated. Nuclei are labeled with DAPI in blue and overexpressed Lpr2E-HA is shown in green, as indicated. LTP accumulates in the fat body cell membranes in the wild type (A). This accumulation is strongly potentiated by the expression of UAS-Lpr2E (C) and is undetectable when a UAS-apoLTPi transgene is co-expressed together with UAS-Lpr2E (E). Lipophorin can be detected in the plasma membrane and in the cytoplasm of wild type fat body cells (B). Expression of UAS-Lpr2E increases lipophorin accumulation in the plasma membrane (D). However, when UAS-apoLTPi is co-expressed together with UAS-Lpr2E, levels of lipophorin in the plasma membrane are similar to the wild type (F), indicating that the increased accumulation of lipophorin induced by Lpr2E requires LTP. (G-H) Imaginal discs of the indicated genotypes shown as controls for Fig 5. UAS-lpr2E and UAS-apoLTPi were expressed in the posterior compartment driven by hh-gal4 for two days. LTP accumulates in the posterior compartment (G). However, additional expression of UAS-LTPi in the fat body driven by Cg-gal4 abolishes LTP accumulation in imaginal discs (H). Note that UAS-Lpr2E is also expressed in the fat body in this genetic combination. Scale bar: 100μm. (TIF) [file pgen.1005356.s009.tif]
